# Overexpression of lily *HsfA3*s in Arabidopsis confers increased thermotolerance and salt sensitivity via alterations in proline catabolism

**DOI:** 10.1093/jxb/ery035

**Published:** 2018-01-31

**Authors:** Ze Wu, Jiahui Liang, Chengpeng Wang, Xin Zhao, Xionghui Zhong, Xing Cao, Guoqing Li, Junna He, Mingfang Yi

**Affiliations:** 1 Beijing Key Laboratory of Development and Quality Control of Ornamental Crops, College of Horticulture, China Agricultural University, Beijing, China; 2 State Key Laboratory for Biology of Plant Diseases and Insect Pests, Institute of Plant Protection, Chinese Academy of Agricultural Sciences, Beijing, China; 3 College of Agronomy, Liaocheng University, Liaocheng, China

**Keywords:** Heat stress transcription factor, heat stress, lily, proline, salt stress, thermotolerance

## Abstract

Although HsfA3 (heat-stress transcription factor A3) is well characterized in heat stress, its roles in other abiotic stresses are less clear. In this study, we isolated two homologous *HsfA3* genes, *LlHsfA3A* and *LlHsfA3B*, from lily (*Lilium longiflorum*). Both genes were induced by heat stress, but not by salt stress. Overexpressing *LlHsfA3A* in Arabidopsis enhanced its basal and acquired thermotolerance, while overexpressing *LlHsfA3B* just enhanced its acquired thermotolerance. In both cases, overexpressing plants showed hypersensitivity to salt stress, and a lack of sucrose exacerbated this salt sensitivity. Using a transient assay, the opposite effects were observed in lily. Further analysis revealed that either *LlHsfA3A* or *LlHsfA3B* overexpression altered normal proline accumulation. During heat treatments, proline increased in wild-type Arabidopsis plants, but no such increase was detected in transgenic plants that showed better basal or acquired thermotolerance. Under salt stress, proline accumulation was decreased in Arabidopsis and lily with the overexpression of *LlHsfA3A* or *LlHsfA3B*. Proline catabolism was activated by overexpression, and both LlHsfA3A and LlHsfA3B affected proline oxidation via regulation of *AtbZIP11*, *AtbZIP44*, and *AtbZIP53* to activate *AtproDH1* and *AtproDH2* in transgenic Arabidopsis. Taken together, our results suggested that overexpression of *LlHsfA3A* or *LlHsfA3B* caused opposite effects on heat and salt tolerance, which may implicate proline catabolism.

## Teaser

Overexpression of lily *HsfA3*s in Arabidopsis leads to changes in proline metabolism mediated via alterations in the regulation of specific bZIP transcription factors and confers increased thermotolerance and salt sensitivity.

## Introduction

All organisms sense temperatures above the normal optimum as heat stress (HS), which can disturb cellular homeostasis and cause many adverse growth and developmental effects, and may even lead to death (Schöffl *et al.*, 1998; Wang *et al.*, 2004; Kotak *et al.*, 2007). To tolerate and survive HS, organisms must activate a heat-stress response (HSR) to alleviate potential damage (Baniwal *et al.*, 2004). In eukaryotic organisms, heat-stress transcription factors (Hsfs) are assumed to play a central role in HSR, by inducing the accumulation of heat-shock proteins (Hsps) and by mediating the activation of other heat-responsive genes involved in cell protective mechanisms and the homeostasis of reactive oxygen species (ROS) (Åkerfelt *et al.*, 2010; Liu *et al.*, 2011; Ohama *et al.*, 2017). As sessile organisms, plants cannot escape high temperature; instead, they have evolved more complex regulatory mechanisms for Hsfs to avoid damage (Nover *et al.*, 2001; Kotak *et al.*, 2007).

Although higher plants have many Hsfs, they share a conserved modular structure. They all contain a DNA-binding domain (DBD), an oligomerization domain (OD or HR-A/B region), and a nuclear localization signal (NLS); some also contain a nuclear export signal (NES). DBDs recognize and bind to heat-stress elements (HSEs) and ODs are necessary for oligomerization. Based on their structural characteristics, Hsfs are allocated into three major classes, A, B, and C. Generally, class-A Hsfs have multiple acidic motifs (AHAs) at the C-terminus and function as transcriptional activators. Several class-B Hsfs contain a tetrapeptide -LFGV- in the C-terminus, which is assumed to function as a repressor motif by interaction with an unknown co-repressor (Nover *et al.*, 2001; Scharf *et al.*, 2012). The function of class-C Hsfs remains unclear; studies in rice and wheat suggest that some also show transcriptional activity (Xue *et al.*, 2014; Hu *et al.*, 2018). Many Hsfs have been characterized in model plants and show considerable functional diversification, being able to play individual roles in complex regulatory networks (von Koskull-Döring *et al.*, 2007). This complex regulation of gene expression is thought to be important, as it enables flexible responses in plants to not only HS but also other stresses and developmental events (Yoshida *et al.*, 2011).

Many studies have shown that most class-A Hsfs play a positive role under different abiotic stresses. In Arabidopsis, mutating all four *AtHsfA1* genes reduces salt and osmotic tolerance (Liu and Charng, 2013). Arabidopsis *AtHsfA2* knockout plants are sensitive to heat, high light, oxidative stress, and anoxia, whereas plants overexpressing *AtHsfA2* show not only greater levels of thermotolerance, but also increased resistance to salt and osmotic stress (Ogawa *et al.*, 2007; Yokotani *et al.*, 2008), oxidative stress (Zhang *et al.*, 2009), and anoxia (Banti *et al.*, 2010). Overexpression of *AtHsfA4a* in Arabidopsis can ameliorate its growth under salt stress (Pérez-Salamó *et al.*, 2014). Overexpression of the ortholog *HSFA4a* in rice and wheat improves their tolerance to cadmium (Shim *et al.*, 2009). Overexpressing *AtHsfA6a* in plants enhances their tolerance to salt and drought stress, and AtHSFA6b, a paralog of AtHsfA6a, operates as a positive regulator participating in ABA-mediated salt and drought resistance (Hwang *et al.*, 2014; Huang *et al.*, 2016). Overexpression of *OsHsfA7* in rice enhances its salt and drought tolerance (Liu *et al.*, 2013). Other HsfAs, such as AtHsfA8, act as sensors of ROS and hence play a role in resistance to oxidative stress (Davletova *et al.*, 2005).

Unlike these other class-A Hsfs, the role of HsfA3 in other abiotic stresses is obscure. AtHsfA3 is reportedly essential for establishing thermotolerance in Arabidopsis; it is one of only two transcription factors (AtDREB2B is the other) whose induction appears to be unique to thermotolerant plants (Larkindale and Vierling, 2008). Under HS, *AtHsfA3* expression depends on AtDREB2A (Schramm *et al.*, 2008); AtDREB2A participates in drought and salt stress responses, and an overexpression of *AtDREB2A* can enhance tolerance to both dehydration and salt (Sakuma et al., 2006*a*, 2006*b*). However, *AtHsfA3* expression is not particularly influenced by high levels of AtDREB2A under drought stress. Efforts have been made to understand this phenomenon. Sato *et al.* (2014, 2016) found that AtDREB2A interacted with a heat-inducible nuclear factor Y (NF-Y), subunit c 10 (NF-YC10/DPB3-1), to form a transcriptional complex that activated *AtHsfA3* expression, and that different NF-Y factors contributed to the target gene selectivity of AtDREB2A under different stress conditions. These results indicate that HsfA3 may not function as an activator in dehydration and salt stress responses. Another study confirmed that AtHsfA6b serves as a direct regulatory factor of AtDREB2A to activate *AtHsfA3*, suggesting it plays a positive role in drought and salt tolerance (Huang *et al.*, 2016). In addition, ectopic-overexpression of *SlHsfA3* (a *HsfA3* from tomato) increases the thermotolerance of transgenic Arabidopsis, but decreases its salt tolerance during the germination stage; it seems that the homologous *HsfA3* may operate as a negative regulator in response to salt stress (Li *et al.*, 2013). Collectively, these findings raise two key questions: what is the actual role of HsfA3 in salt stress, and why can it play opposite roles under heat and salt stress?

Lily is an important horticultural crop with poor thermotolerance, and hence by studying its HSR mechanism we can aim to improve its germplasm. Given the demonstrated importance of Hsfs, we have focused on lily’s Hsfs pathway (Xin *et al.*, 2010; Gong *et al.*, 2014). Here, we identify two *LlHsfA3* genes from lily that positively regulate thermotolerance but negatively regulate salt tolerance. Going further, we also demonstrate that these different tolerant behaviors are associated with the proline-mediated resistance pathway.

## Materials and methods

### Plant material and growth conditions

Two commercial cultivars of lily, ‘White heaven’ (*Lilium longiflorum*) and ‘Siberia’ (*Lilium* Oriental hybrids), were studied and their thermotolerances were determined following the methods of Xin *et al.* (2010). Lily plantlets were cultured on Murashige and Skoog (MS) medium at 22 °C in a standard culture room with a 16-h light/8-h dark photoperiod. To analyse the functions of *LlHsfA3A* and *LlHsfA3B*, we selected *Arabidopsis thaliana* (Col-0) as a testing platform, since its genetic transformation methods are well established. The growth conditions of the Arabidopsis plants were the same as those described by Gong *et al.* (2014).

### Full-length cloning and sequence analysis of *LlHsfA3A* and *LlHsfA3B*

After a heat treatment of 37 °C for 3 h, total RNA of ‘White heaven’ leaves was extracted with a RNAprep Pure Plant Kit (Tiangen, China). First-strand cDNA was synthesized using the M-MLV reverse transcriptase (TaKaRa, Japan), with an oligo dT primer. Following the method of homology-based cloning, a conserved partial sequence of the *LlHsfA3* cDNA was amplified using degenerate primers (forward, TCAAGCACAACAAYTTCTCCAGC; reverse, CTTGGCCARGAAHGAGACCA) based on the DNA-binding domain of variant HsfA3s, then cloned into pMD-18T (TaKaRa, Japan) for sequencing, and two distinguishing fragments were acquired. The RACE technique was used for rapid amplification of cDNA ends with a 5′- and 3′-one-step Full Race kit (Takara, Japan). After sequencing, two full-length sequences of the *LlHsfA3*s (*LlHsfA3A* and *LlHsfA3B*) were obtained and then translated into amino acids using ExPASy (http://web.expasy.org/translate/). The RT-PCR method was used to detect the *LlHsfA3*s genes in the different cultivars (for primers see Supplementary Table S1 at *JXB* online). The conserved domains were identified using the Heatster web server (http://www.cibiv.at/services/hsf/). Phylogenetic relationships were analysed using the ClustalW 2.0 and MEGA 5.0 software.

### Promoter isolation

Genomic DNA was extracted from ‘White heaven’ leaves using a Plant Genprep DNA kit (Zomanbio, China). The promoters of *LlHsfA3A* and *LlHsfA3B* were cloned using the hiTAIL-PCR method (Liu and Chen, 2007). The 1200-bp upstream fragments from the start ATG of *LlHsfA3A* and *LlHsfA3B* were isolated and identified.

### Plasmid construction

The *pCAMBIA1300* vector was used for stable transformation. *LlHsfA3A* and *LlHsfA3B* were amplified by primers with *Sal*I and *Kpn*I sites, and then cloned into *pCAMBIA1300* to construct the vectors *p1300-LlHsfA3A* and *p1300-LlHsfA3B*. *p1300-GFP-C* and *p1300-GFP-N* were used for subcellular localization. The same amplified fragments were inserted into the *Sal*I/*Kpn*I sites of the vector *p1300-GFP-N* to produce the N-terminal fusion of green fluorescent protein (GFP). *LlHsfA3A* and *LlHsfA3B* were amplified with the same restriction sites, but without a termination codon, then cloned into *p1300-GFP-C* to produce the C-terminal fusion of GFP. All these reconstructed genes were driven by a 35S promoter. The *pCAMBIA1391* vector containing *GUS* (*β-glucuronidase*) served as the transgenic vector to assay promoter activation. A 1200-bp region of genomic DNA upstream of the ATG of *LlHsfA3A* and *LlHsfA3B* was amplified individually from the genome of lily using primers with *Pst*I and *Sma*I sites, and then cloned into *pCAMBIA1391*. The *pGBKT7* vector was used for transcriptional activity analysis. The full-length *LlHsfA3A* (1–1647) and *LlHsfA3B* (1–1527) were amplified by PCR and cloned between the *EcoR*I and *Sal*I sites of *pGBKT7* to construct the vectors *pBD-LlHsfA3A* and *pBD-LlHsfA3B*. For the deletion assay, the C-terminal-truncated fragments of *LlHsfA3A* (1–1374) and *LlHsfA3B* (1–1260) were PCR-amplified from *pBD-LlHsfA3A* and *pBD-LlHsfA3B* and cloned into the *EcoR*I/*Sal*I sites of *pGBKT7*, producing the vectors *pBD-LlHsfA3Ad* and *pBD-LlHsfA3Bd*, respectively. For the mutation assay, the positions of 1399–1401 (TGG), 1453–1455 (TGG), 1516–1518 (TGG), and 1573–1575 (GTT) of *LlHsfA3A*, and the positions of 1285–1287 (TGG) and 1339–1341 (TGG) of *LlHsfA3B* were replaced with GGG. These mutations were introduced via gene synthesis (BGI, China) and cloned into *pGBKT7* as before, to produce the *pBD-LlHsfA3Am* and *pBD-LlHsfA3Bm* vectors. The primers designed for plasmid construction are listed in Supplementary Table S2.

### Subcellular localization and transcriptional activity analyses

The methods used for these analyses of *LlHsfA3A* and *LlHsfA3B* are described by Gong *et al.* (2014).

### Abiotic stress treatments and gene expression assay of lily

Two-week-old healthy lily plantlets (in bottles, diameter 6 cm, height 12 cm) of uniform size (bulb perimeter 1.5–2.0 cm, number of leaves 3–5, height 6–8 cm) were selected for the treatments. To analyse the temperature response of expression patterns, the selected plants were exposed to different temperatures (16, 22, 28, 32, 37, 42 °C) for 3 h, or to different durations of HS (0, 1, 3, 6, 12, 24, 48 h) at 37 °C. For salt and mannitol treatments, plants were removed from the growth medium and transferred to 200 mM NaCl or 300 mM mannitol solution for 24 h at 22 °C (deionized water control). All treatments were applied in a temperature-controlled incubator (SUMSUNG, DRP-9082, China) without light. Following treatment, leaves were collected and frozen immediately in liquid nitrogen. Total RNA was extracted as described above, and reverse transcription was performed with a HiScript II kit (Vazyme, China). Real-time quantitative PCR (qPCR) was used to determine the expression levels (following the method of Gong *et al.*, 2014). The *18S rRNA* of lily served as a quantifying control. Primers designed for the qPCR analysis are listed in Supplementary Table S3.

### Stable transformation of Arabidopsis

Recombinant vectors of *pCAMBIA1300* and *pCAMBIA1391-GUS* were transformed into 5-week-old Arabidopsis plants (col and SALK_011131) using the floral-dip method (Clough and Bent, 1998). Homozygous mutant SALK_011131 plants were selected and the lack of *AtHsfA3* expression was verified by RT-PCR (for primers see Supplementary Table S4). Transformed seeds were selected on MS medium containing 30 mg l^−1^ hygromycin. All transgenic lines were identified by qPCR; three T_3_-generation homozygous lines were selected for the gene functional analysis detailed below.

### GUS activity assay of promoter transgenic lines

Histochemical staining for the GUS activity assay in the transgenic plants followed the methods of Hwang *et al.* (2014). The 7-d-old seedlings were immersed in the staining solution and incubated at 37 °C for 12 h. Salt and mannitol treatments lasted 12 h, and HS lasted 3 h at 37 °C. Chlorophyll was cleared from the plant tissues by immersing them in 70% ethanol for 24 h. GUS activity was measured as described by Xu *et al.* (2006).

### Abiotic stress treatments of transgenic Arabidopsis

Arabidopsis seeds were sterilized with 1.0% (v/v) NaClO for 15 min, then washed five times with sterile water, and sown onto MS medium. After vernalization for 3 d at 4 °C in the dark, plants were then grown at 22 °C under a 16-h light/8-h dark regime, in a standard culture room. For HS, plates containing 5-d-old seedlings were sealed with plastic electrical tape and transferred to an incubator (SUMSUNG, DRP-9082, China) (temperatures are indicated on the relevant figures in the Results). After HS, the less-thermotolerant seedlings would lose their green color and die, and their survival rate was recorded with a 7-d-recovery period.

To investigate salt and mannitol effects on germination, wild-type and transgenic seeds were sown onto MS medium containing salt (0, 50, 100, 150 mM) or mannitol (0, 100, 200, 400 mM) and their germination was recorded daily. To investigate the effects of salt, mannitol, and sucrose on root growth, 6-d-old seedlings were transferred to 1/2-MS medium containing salt (0, 120 mM) or mannitol (0, 200, 300 mM) with a high (2%) or low (0.5%, 0%) concentrations of sucrose. Root length was recorded initially and 7 d later to quantify root elongation. Seedlings at 10 d old were placed on filter paper and treated with 1/2-MS liquid medium with 150 mM NaCl, and 7-d-old seedlings were likewise treated with 300 mM mannitol; after 7 d, the phenotypes were recorded. The 10-d-old seedlings were transplanted to plastic cups (180 cm^3^) containing a sterile rooting mixture (peat/vermiculite, 1/1) and cultured at 22 °C under a 16-h light/8-h dark regime in a standard culture room. After 3 weeks of growth, a 400-mM NaCl or mannitol solution was applied three times (every 5 d); the phenotypes were photographed. Leaves of the mannitol-treated plants were cut and physiological indexes were determined (water and anthocyanin contents, ion leakage).

### Proline measurement

The free proline content was measured at appropriate times using a colorimetric assay (Bates *et al.*, 1973). To determine the proline content in Arabidopsis plants at different stages of development, 7-d-old seedlings were transferred to fresh MS medium for vertical culturing, and after 3 d and 7 d the whole plant was sampled. The preparation of HS samples is detailed in the relevant figures in the Results. For samples under salt and mannitol stress, 7-d-old seedlings were transferred to 1/2-MS medium (with or without sucrose) and treated with 150 mM NaCl or 300 mM mannitol; they were vertically cultured for 72 h, then the whole plant was sampled.

### Proline treatment of transgenic Arabidopsis

Seeds were sown onto MS medium supplemented with 10 mM proline and 150 mM NaCl; their germination rate was recorded daily. The 5-d-old seedlings grown on proline-supplemented MS medium were exposed to HS (see figures in Results for details), and after a 7-d-recovery period their survival rate was recorded.

### Gene expression assay of transgenic Arabidopsis

Following treatment, plants were collected and total RNA was extracted for gene expression analysis (qPCR method). Genes involved in the proline metabolic pathway and HSR pathway were detected (for primers see Supplementary Table S3). For the HS treatment, 5-d-old seedlings were treated as described above; for salt or mannitol stress, 6-d-old seedlings were transferred to 1/2-MS liquid medium (with or without sucrose) and treated with 150 mM NaCl or 300 mM mannitol for 12, 24, or 48 h. For the control (0 h), seedlings were transferred to 1/2-MS liquid medium containing 2% sucrose for 12 h.

### Transient expression and abiotic treatments in lily petal discs

The *p1300-GFP-C*, *p1300-LlHsfA3A*, and *p1300-LlHsfA3B* vectors were transformed into the *Agrobacterium tumefaciens* strain GV3101. Their cultures were harvested by centrifugation, re-suspended in an infiltration buffer (10 mM MgCl_2_, 200 mM acetosyringine, 10 mM MES, pH 5.6) to a final OD_600_ of about 1.0, and placed in the dark at room temperature for 5 h before vacuum infiltration. For transient expression, a 6-cm length of unopened flower buds of ‘Siberia’ was selected; the outer petals were removed, and 1-cm-diameter discs were excised from the inner petals using a hole-puncher (eight per petal). Discs were immersed in a bacterial suspension and infiltrated under a vacuum at 0.7 MPa. After release of the vacuum, the discs were washed with deionized water and put on a semi-solid plate (0.4% agar) at room temperature for 48 h in the dark. qPCR was used to determine expression levels in these discs. For the HS treatment, after 48 h the discs were treated at 42 °C for 1 h, then harvested immediately, and their proline content determined (including untreated discs as controls). For the salt treatment, half of the discs were transferred to a fresh plate and the rest to a plate supplemented with NaCl (200 mM NaCl). After 24 h, these discs were harvested to determine their proline content, and to measure their relative ion leakage.

### Chromatin immunoprecipitation assay (ChIP)

As described above, LlHsfA3A and LlHsfA3B were cloned into the *p1300-GFP-C* vector to produce GFP fusions. The constructs were transformed into Arabidopsis plants, resulting in LlHsfA3A-GFP-ox and LlHsfA3B-GFP-ox plants. The 10-d-old transgenic seedlings grown on MS medium were used for ChIP assays, as described by Fode and Gatz (2009). An anti-GFP antibody was used for immunoprecipitation (Sigma). DNA was purified using Wizard SV Gel and the PCR Clean-Up System (BioTeke, China). ChIP samples were amplified by RT-PCR (for primers see Supplementary Table S5).

## Results

### Isolation of two *HsfA3* homologous genes

**Fig. 1.**
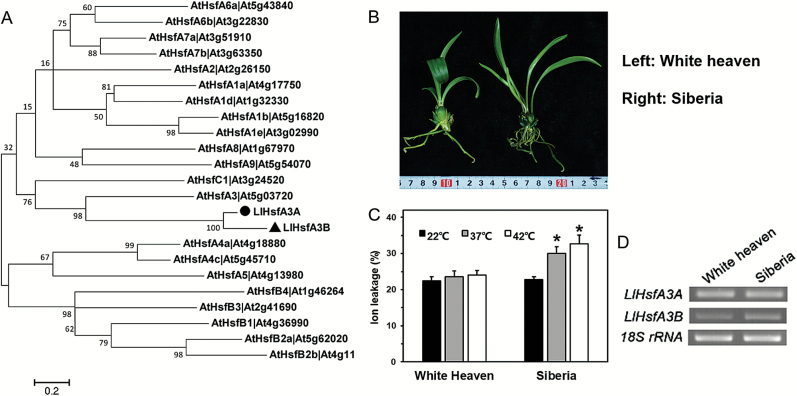
Phylogenetic analysis of the LlHsfA3A and LlHsfA3B proteins and RT-PCR detection of *LlHsfA3A* and *LlHsfA3B* in different lily cultivars. (A) Phylogenetic tree of LlHsfA3A and LlHsfA3B proteins and the Arabidopsis Hsf family. The amino acid sequences of the Arabidopsis Hsfs were downloaded from the TAIR website (www.arabidopsis.org). The software MEGA 5.0 was used to reconstruct the evolutionary tree. Node values are percentages of bootstraps generated with 1000 replicates. The scale bar shows an evolutionary distance corresponding to 0.2 amino acid substitutions per site. (B) Example plants of the two lily cultivars, ‘White heaven’ and ‘Siberia’. (C) Relative ion leakage of the leaves after heat stress (HS) at 37 or 42 °C for 1 h compared with the control (22 °C). Each treatment included three plants, and data are means (±SD) of three independent experiments. Significant differences compared with the control with determined using Student’s *t*-test (*P<0.05). (D) Detection of *LlHsfA3A* and *LlHsfA3B* in the HS transcripts of ‘White heaven’ and ‘Siberia’ using RT-PCR (32 cycles). These bands were confirmed by sequencing.

Two *HsfA3* homologous genes were isolated from lily: *LlHsfA3A* and *LlHsfA3B*. They were predicted to encode proteins containing 548 and 508 amino acids (a.a.), respectively. Phylogenetic analysis (using all 21 Arabidopsis Hsfs) revealed that LlHsfA3A and LlHsfA3B were closely related to AtHsfA3, indicating they were homologs (Fig. 1A). We then determined the *HsfA3*s in two distinct thermotolerance cultivars: ‘White heaven’, with less ion leakage after HS, showed more tolerance than did ‘Siberia’. RT-PCR was used to analyse the transcripts in them, and both *LlHsfA3* genes were detected (Fig. 1B–D).

### Subcellular localization and transcriptional activity analyses of *LlHsfA3A* and *LlHsfA3B*

**Fig. 2.**
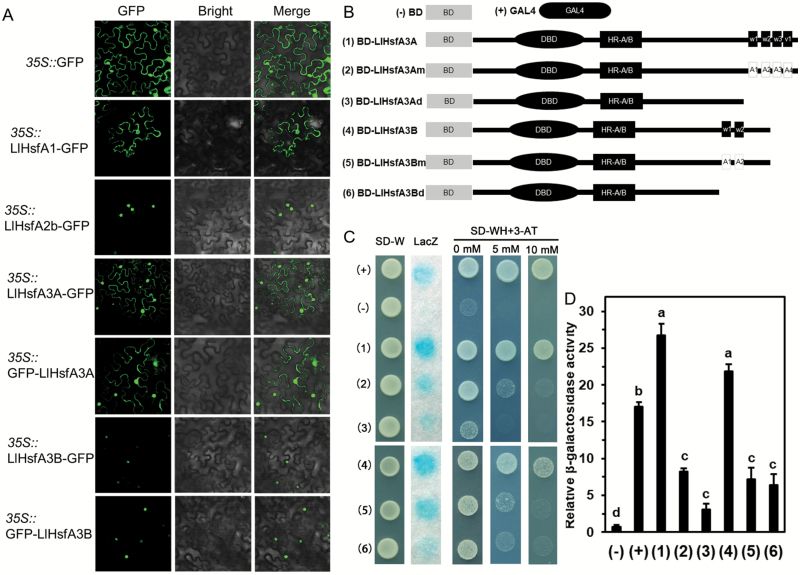
Subcellular localization and transactivation assays of the LlHsfA3A and LlHsfA3B proteins. (A) Transient expression profiles of LlHsfA3A and LlHsfA3B in tobacco leaves. Confocal microscopy of tobacco leaf cells transfected with LlHsfA3A and LlHsfA3B fused to the N- or C-terminal GFP reporter gene, controlled by the 35S promoter. The empty GFP vector served as the negative control. LlHsfA1-GFP and LlHsfA2b-GFP served as the positive controls (Gong *et al.*, 2014; Xin *et al.*, 2017). (B) Six constructs were used for the transactivation assay. For the mutation assay of the AHA motifs, the a.a. sites 467(W1), 485(W2), 506(W3), and 525(V1) of LlHsfA3A were replaced by alanine (2); likewise, the a.a. sites 429(W1) and 447(W2) of LlHsfA3B were replaced by alanine (5). For the deletion assay, the activation domain from position 456 (3) of LlHsfA3A and position 427 (6) of LlHsfA3B were truncated. BD and GAL4 served as the negative and positive controls, respectively. (C) Transactivation activity of the different constructs in yeast. The SD-Trp medium detected transformation, the SD –Trp/–His medium with or without 3-AT examined the transformants’ growth, and X-gal staining detected the β-galactosidase activity of transformed yeast cells. Over five yeast cells were tested in each construct. One representative image of them is shown. (D) The β-galactosidase activity was measured via an enzyme assay. Data are means (±SD) of three independent experiments (three yeast lines measured in each independent experiment). Different letters indicate significant differences among the different transformants (Student–Newman–Keuls test, *P*<0.05).

Both LlHsfA3A and LlHsfA3B had a structure typical of class-A Hsfs. When they were subjected to a Blast search with other HsfA3s from *Arabidopsis thaliana*, *Oryza sativa*, *Phoenix dactylifera*, and *Solanum lycopersicum*, the amino acid parts of the DBD, HR-A/B, and NLS domains were quite similar to the HsfA3s from these species (Supplementary Fig. S1). Transient expression of GFP in tobacco leaves, either fused at the N- or the C-terminal, showed that LlHsfA3B was stably distributed in the nucleus, whilst LlHsfA3A was localized in the nucleus and cytoplasm (Fig. 2A).

Based on domain prediction, the activation domain of LlHsfA3A was located at a.a. sites 423–525, with four typical units of AHA motifs (463–469, 482–487, 502–511, 519–525), while the activation domain of LlHsfA3B was located at a.a. sites 425–449, with two typical units (425–431, 444–449) (Supplementary Fig. S1). We then considered whether these sites were necessary for transactivation. The required *pGBKT7* vectors were constructed (Fig. 2B) and transformed into yeast AH109. Full-length LlHsfA3A and LlHsfA3B showed a strong transactivation activity in the yeast cells. After deleting the C-terminal activation domain of LlHsfA3A and LlHsfA3B, or mutating the core a.a. residues of their AHA motifs, their transactivation activity decreased significantly (Fig. 2C). On the other hand, the β-galactosidase activity in transformed yeast was similar between the deletion and mutation assays (Fig. 2D), which indicated that the predicted AHA motifs conferred the activation potential of LlHsfA3A and LlHsfA3B.

### *LlHsfA3A* and *LlHsfA3B* expression in response to environmental stress

**Fig. 3.**
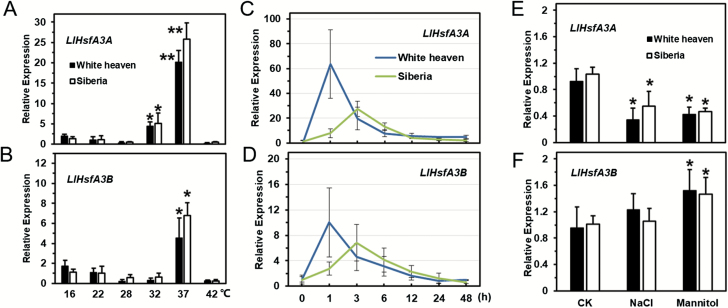
Expression patterns of *LlHsfA3A* and *LlHsfA3B* genes in lily leaves under different abiotic stress treatments. Two cultivars, ‘White heaven’ and ‘Siberia’, were investigated. (A, B) Leaf samples were collected after 3 h under different ambient temperature treatments. (C, D) Leaf samples were collected after different periods of exposure to 37 °C. (E, F) Tissue-cultured seedlings of lily with roots were treated with water (control, CK), salt solution (NaCl, 200 mM), or mannitol solution (400 mM) for 24 h, after which their leaves were collected. The data were normalized to *18S rRNA* of lily, and the 2^–ΔΔ*C*t^ method was used in the qPCR analysis. Each treatment included three plants. Data are means (±SD) of three independent experiments. Significant differences were determined using Student’s *t*-test (*P<0.01, **P<0.001), compared with 22 °C in (A, B) and compared with the control in (E, F).

Compared with 22 °C, *LlHsfA3A* expression was enhanced after 3 h of treatment at 32 °C and 37 °C. At 32 °C, *LlHsfA3A* expression increased 4.2-fold in ‘White heaven’ and 6.1-fold in ‘Siberia’, while at 37 °C its expression was up-regulated dramatically in both cultivars, by about 20-fold in ‘White heaven’ and 26-fold in ‘Siberia’ (Fig. 3A). On the other hand, *LlHsfA3B* was only induced at 37 °C, with its expression being up-regulated 4.5-fold in ‘White heaven’ and 6.8-fold in ‘Siberia’ (Fig. 3B). At 37 °C, *LlHsfA3A* and *LlHsfA3B* were rapidly induced in ‘White heaven’, with peaks in expression being observed for the 1-h treatment, after which expression gradually decreased. In contrast to ‘White heaven’,

the expression of *LlHsfA3A* and *LlHsfA3B* in ‘Siberia’ peaked after the 3-h treatment. Generally, in both cultivars, the expression of *LlHsfA3A* and *LlHsfA3B* showed similar trends under HS, but the relative change of *LlHsfA3B* was lower than that of *LlHsfA3A* (Fig. 3C, D). In both ‘White heaven’ and ‘Siberia’, *LlHsfA3A* expression was inhibited by the salt and mannitol treatments, but *LlHsfA3B* expression was slightly induced by mannitol (Fig. 3E, F).

We also analysed the promoter activity of *LlHsfA3A* and *LlHsfA3B*. GUS activity of transgenic seedlings showed that the promoters had basal activity, but this was greatly elevated by HS in the leaves and roots. However, the promoter activity of LlHsfA3A and LlHsfA3B was hardly influenced by the salt and mannitol treatments (Supplementary Fig. S2).

### *LlHsfA3A* and *LlHsfA3B* play distinct roles in thermotolerance of transgenic Arabidopsis

**Fig. 4.**
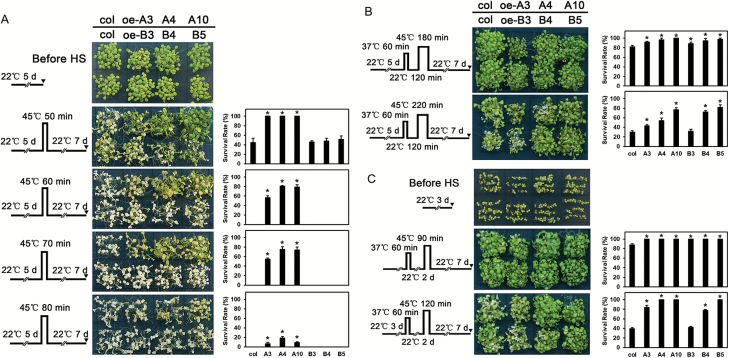
Thermotolerance analysis of transgenic plants. Three LlHsfA3A overexpression lines (oe-A3, oe-A4, oe-A10) and three LlHsfA3B overexpression lines (oe-B3, oe-B4, oe-B5) were used in this experiment. The phenotypes of the wild-type and transgenic seedlings are shown following their treatment with different heat stress (HS) regimes (A–C), which are indicated to the left of the images. (A) The 5-d-old seedlings were directly exposed to 45 °C to detect basal thermotolerance (BT). (B) The 5-d-old seedlings were first treated with a non-lethal temperature of 37 °C for 60 min, followed by recovery for 2 h at 22 °C, and then subjected to 45 °C to detect acquired thermotolerance after short-time recovery (ATSR). (C) The 3-d-old seedlings were treated with 37 °C for 60 min, then cultured under 22 °C for 2 d, then exposed to 45 °C to detect acquired thermotolerance after long-time recovery (ATLR). The plants in (A–C) were photographed 7 d after the final HS. The survival rate of both the wild-type and transgenic lines was measured at day 7 after the final HS treatment. Data are means (±SD) of three independent experiments. One representative set of plants is shown for each experiment. Each treatment included over 30 seedlings of each line. Significant differences between the wild-type and transgenic plants are indicated (*P<0.05, Student’s *t*-test).

*LlHsfA3A* and *LlHsfA3B* transgenic Arabidopsis lines were identified by qPCR, and the positive lines oe-A3, oe-A4, oe-A10, oe-B3, oe-B4, and oe-B5 were subjected to further study (Supplementary Fig. S3). Following Charng *et al.* (2006), different HS patterns were designed for detection of thermotolerance. Seedlings were directly exposed to 45 °C to detect their basal thermotolerance (BT); the oe-A lines showed better BT with a higher survival rate under the different HS duration conditions, whereas the oe-B lines did not show an improved BT (Fig. 4A). As shown by the detection of acquired thermotolerance after short-time recovery (ATSR) Fig. 4B), under a treatment of 45 °C for 180 min- the survivorship of both the oe-A and oe-B lines exceeded that of the wild-type. Extending the HS duration to 220 min, the oe-A lines, and oe-B4 and oe-B5 still grew better than the wild-type. With regards to acquired thermotolerance after long-time recovery (ATLR) (Fig. 4C), the transgenic lines showed no impaired survival when treated at 45 °C for 90 min, and the survival of the oe-A lines, and oe-B4 and oe-B5 was still better than in the wild-type after 120 min duration of HS. These results demonstrated that *LlHsfA3A* overexpression could improve the BT and acquired thermotolerance (AT) of Arabidopsis, whereas *LlHsfA3B* overexpression improved AT only.

The *hsfa3* homozygous mutant line SALK_011131 was identified, in which *LlHsfA3A* and *LlHsfA3B* were then individually overexpressed; using qPCR assays, the complemented lines A/*hsfa3-1*, *2*, *6*, and B/*hsfa3-1*, *2*, *5* were selected for subsequent detection (Supplementary Fig. S4). After the BT treatment, more mutant than wild-type seedlings died, while the BT of A/*hsfa3*s was restored to the wild-type level; however, the BT of the B/*hsfa3*s did not differ significantly from that of the mutant. After AT treatment, the mutant showed poor AT, but all the complemented lines showed AT comparable with the wild-type (Supplementary Fig. S4). These results indicated that LlHsfA3A alone could compensate for the function of AtHsfA3, whereas LlHsfA3B could only partly compensate for the AT function of AtHsfA3. Although LlHsfA3A and LlHsfA3B played distinct roles in thermotolerance in transgenic Arabidopsis, some same-target genes of AtHsfA3 were commonly induced; for example, apart from *AtGolS4* not being induced in oe-B4, *AtGolS1*, *AtGolS2*, *AtHsp22.0*, *AtHsp25.3*, *AtHsp19.9*, and *AtHsp70b* were induced in both oe-A4 and oe-B4 (Supplementary Fig. S5). This result indicated that similar and diverse regulation networks operate between LlHsfA3A and LlHsfA3B.

### Overexpression of *LlHsfA3A* or *LlHsfA3B* impairs salt tolerance of transgenic Arabidopsis

**Fig. 5.**
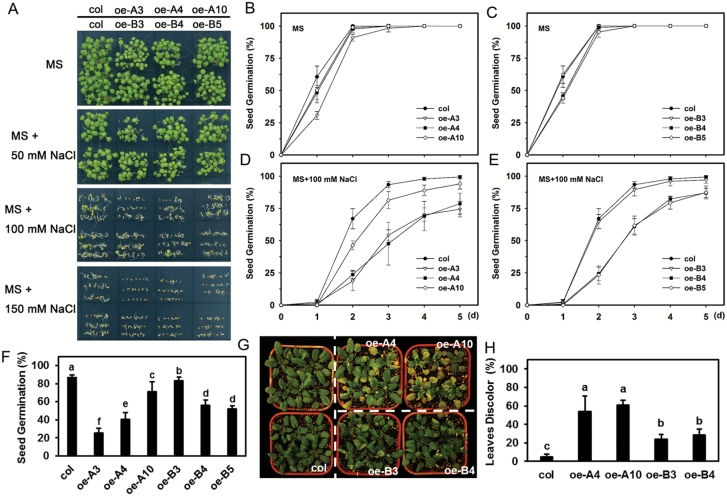
Seed germination and seedling growth of transgenic plants in response to salt stress. (A) Seed germination on NaCl-supplemented MS plates after 5 d under light. One representative image of three independent experiments is shown (each treatment included at least 30 seeds of each line). (B–E) Seeds were sowed on MS medium with or without 100 mM NaCl and the germination rate was recorded daily. (F) Seeds were sowed on MS medium with 150 mM NaCl, and the percentage germination was recorded after 5 d. Data represent the means (±SD) of three independent experiments. More than 30 seeds of each line were tested in each treatment. Different letters indicate significant differences among the lines (Student–Newman–Keuls test, *P*<0.05). (G) Plants were grown in soil for 3 weeks, and then irrigated with a NaCl solution (400 mM) for 15 d. An image from one representative result is shown. (H) The proportion of discoloration (%) of all plant test lines in (G) were calculated. Data are means (±SD) of three independent experiments. Over 18 plants of each line were examined in each independent experiment. Different letters indicate significant differences among the lines (Student–Newman–Keuls test, *P*<0.05).

Under either normal conditions or when treated with 50 mM NaCl, seed germination did not differ between the transgenic lines and the wild-type (Fig. 5A–C). However, when the concentration was doubled to 100 mM, the germination of the oe-A lines, and oe-B4 and oe-B5 was markedly repressed (Fig. 5D, E). When the NaCl concentration was increased to 150 mM, the 5-d germination rate of all the transgenic lines was reduced (Fig. 5F), With the exception that, not surprisingly, the germination of SALK_011131 was better than that of the wild-type and complemented lines under salt stress (Supplementary Fig. S6). For soil-grown plants irrigated with NaCl solution, the oe-A and oe-B lines were more susceptible to NaCl, as evidenced by their leaf discoloration being more severe than that seen in the wild-type (Fig. 5G, H). These results suggested that overexpression of *LlHsfA3A* or *LlHsfA3B* in Arabidopsis would impair its salt tolerance, and that HsfA3 may negatively regulate the salt response.

### Lack of sucrose aggravates salt sensitivity of transgenic Arabidopsis

**Fig. 6.**
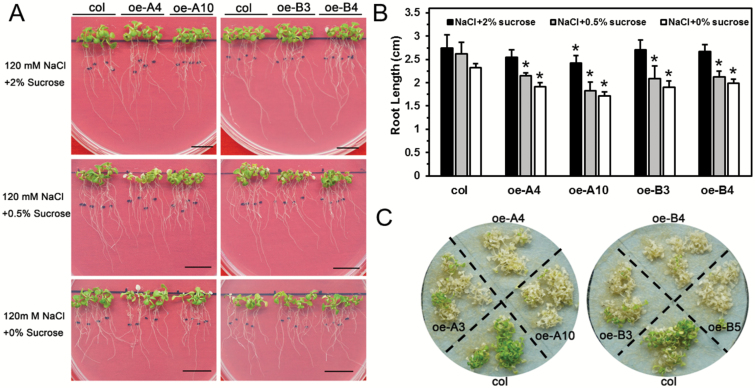
Lack of sucrose affected the salt tolerance of transgenic plants. (A) Images of 6-d-old seedlings after 7 d of growth. Scale bars are 1 cm. (B) The root elongation of each plant was measured, and the average value for was determined for each line across three independent experiments (each experiment included 12 plants of each line). Data are means (±SD) of three independent experiments. Significant differences compared with the wild-type were determined using Student’s *t*-test (*P<0.05). (C) The 10-d-old seedlings were transferred to filter paper and treated with 150 mM NaCl. Images were taken after 7 d. One representative image of three independent experiments is shown (each experiment included at least 30 plants).

There was no significant difference in root elongation between the wild-type and transgenic lines at the post-germination stage, either with or without NaCl in the MS medium, except for oe-A10 (Supplementary Fig. S7, 6A, B). However, with lower levels of sucrose in the medium, root growth of the oe-A and oe-B plants under salt stress was significantly reduced compared with that of the wild-type (Fig. 6A, B). For seedlings on filter paper treated with NaCl solution, almost all of the oe-A and oe-B plants were completely discolored after 7 d, and the salt-sensitive phenotype was more apparent than in the wild-type (Fig. 6C). This result suggested that a lack of sucrose could aggravate salt sensitivity of the oe-A and oe-B lines.

### Overexpression of *LlHsfA3A* or *LlHsfA3B* does not decrease mannitol tolerance in transgenic Arabidopsis

**Fig. 7.**
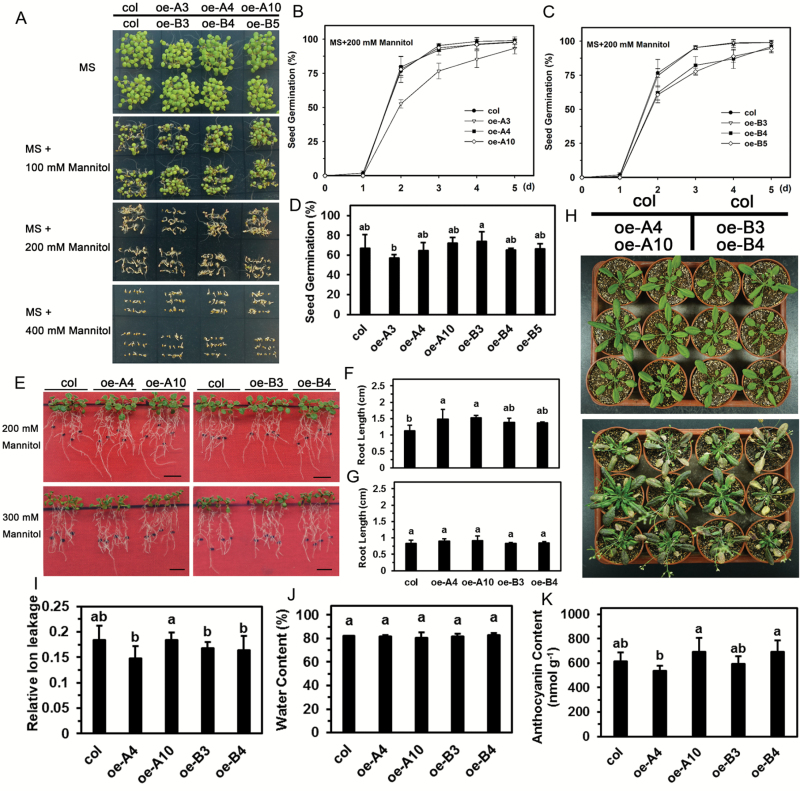
Seed germination and seedling growth of transgenic plants in response to mannitol stress. (A) Seed germination with mannitol on plates after 5 d under light. One representative image of three independent experiments is shown (each treatment included at least 30 seeds of each line). (B–D) Seeds were sown on MS medium with 300 mM mannitol and the germination rate was recorded daily. Data are means (±SD) of three independent experiments (more than 30 seeds of each line were tested in each treatment). (E–G) Seedling root growth was measured and after 7 d. One representative image of three independent experiments is shown (each experiment included 12 plants of each line). Data are means (±SD) of three independent experiments. Different letters indicate significant differences among the lines (Student–Newman–Keuls test, *P*<0.05). (H) Seedlings after treatment with the 400 mM mannitol solution (see Methods for details). One representative image of three independent experiments is shown (each treatment included at least six plants). (I) Relative ion leakage, (J) water content, and (K) anthocyanin content of the leaves of wild-type and transgenic plants after the 400 mM mannitol treatment shown in (H). Data are the means (±SD) of three biological replicates. Different letters indicate significant differences among the lines (Student–Newman–Keuls test, *P*<0.05).

Plants are known to share a similar regulatory pathway for salt and mannitol stress, so we also examined the mannitol tolerance of the transgenic lines. When treated with 100 or 200 mM mannitol, the germination of wild-type and transgenic lines was similar (Fig. 7A–C). Even when mannitol was increased to 400 mM, the germination rate showed no significant differences (Fig. 7D). Remarkably, the root growth of oe-A plants even exceeded that of the wild-type under 200 mM mannitol, but this difference disappeared under 300 mM; oe-B plants grew no differently than the wild-type plants under either 200 or 300 mM mannitol (Fig. 7E–G). We also examined the effect of sucrose on mannitol tolerance and found that a lack of sucrose did not cause root elongation to differ between the wild-type and transgenic lines under mannitol stress, as the phenotypes were similar for seedlings treated with 300 mM mannitol solution on filter paper (Supplementary Fig. S8). For soil-grown plants irrigated with mannitol, all the leaves became purple/red and withered, and their physiological indexes were similar between the transgenic and wild-type lines (Fig. 7H–K). Hence, the overexpression of *LlHsfA3A* or *LlHsfA3B* did not decrease mannitol tolerance in Arabidopsis.

### Thermotolerance function of *LlHsfA3A* and *LlHsfA3B* involves proline metabolic changes

**Fig. 8.**
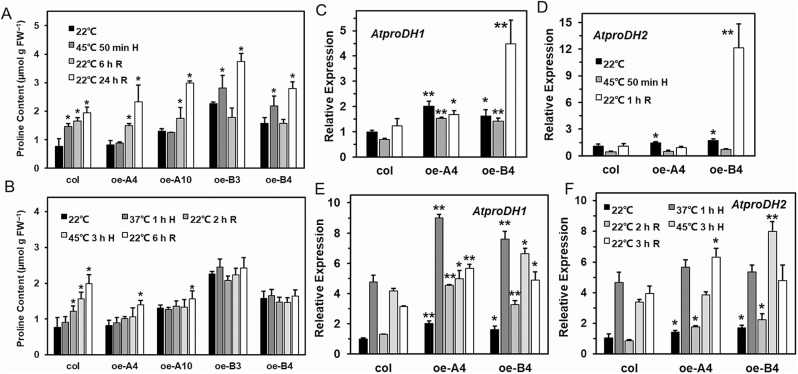
Determination of free proline content and analysis of *AtproDH1* and *AtproDH2* expression in Arabidopsis. Free proline content at different stages treatment for (A) basal thermotolerance (BT) and (B) acquired thermotolerance after short-time recovery (ATSR) using 5-d-old seedlings. H, heat stress; R, recovery. Data are means (±SD) of three independent experiments. Significant differences compared with 22 °C were determined using Student’s *t*-test (*P<0.05). (C–F) Fold-changes in the expression levels of *AtproDH1* and *AtproDH2* were detected at different stages of the BT treatment (C, D) and the ATSR treatment (E, F) using qPCR with 5-d-old seedlings. Data are means (±SD) of three biological replicates. Significant differences between the wild-type and transgenic plants were determined using Student’s *t*-test (*P<0.05, **P<0.01).

Since proline metabolism is conjointly related to sugar, heat, and salt signaling (Kavi Kishor and Sreenivasulu, 2014), we focused on the proline-mediated resistance pathway in the overexpression lines. It was observed that the proline content of transgenic lines at different developmental stages differed from that of the wild-type (Supplementary Fig. S9), and hence overexpression of *LlHsfA3A* or *LlHsfA3B* altered normal proline accumulation. To further understand how *LlHsfA3A* and *LlHsfA3B* might affect proline accumulation, and whether the metabolic changes were related to the discriminative thermotolerance between the oe-A and oe-B lines, the transcript levels of genes of proline metabolism and proline contents at different HS stages were analysed using 5-d-old seedlings. *AtproDH1* and *AtproDH2*, two key enzyme genes of proline degradation that catalyse the oxidation of proline to P5C in mitochondria, were distinctly induced in both oe-A4 and oe-B4 (Fig. 8C, D). *AtP5CS1* is a key enzyme gene for proline synthesis that converts glutamate into P5C, and in oe-B4 its expression was also up-regulated (Supplementary Fig. S10), which may have contributed to the high levels of proline seen under normal conditions (Fig. 8A). Notably, during HS of the BT treatment, *AtproDH1* still sustained a relatively high expression in oe-A4 and oe-B4. In addition, both in oe-A4 and oe-B4 there was an enhanced expression of *AtP5CDH*; AtP5CDH catalyses the second step of proline oxidation in which the P5C is converted to glutamate. Although other proline metabolism-related genes were also influenced in oe-A4 or oe-B4, their changes in expression were rather weak (Supplementary Fig. S10).

With the BT treatment, proline increased gradually in the wild-type after HS, but the content remained steady in the oe-A lines during HS; later, during recovery, proline increased (Fig. 8A). In oe-B4, proline increased during HS much like in the wild-type, but it decreased sharply after a short-term recovery (Fig. 8A), perhaps because *AtproDH1* and *AtproDH2* were highly expressed (Fig. 8C, D). With the ATSR treatment, the wild-type accumulated proline continuously; however, proline fluctuated only slightly in oe-A4 and oe-B4 (Fig. 8B). The expressions of *AtproDH1* and *AtproDH2* were both higher than that of the wild-type during the ATSR-treatment period, which may have conferred proline homeostasis under HS (Fig. 8E, F). In summary, we found that the oe-A and oe-B plants showed better BT or AT that was accompanied with no significant proline increase during HS, and that the induction of genes for proline metabolism, especially those of catabolism, might contribute to the differences in proline accumulation and thermotolerance.

### *LlHsfA3A* and *LlHsfA3B* participate in proline catabolism by regulation of bZip factors

**Fig. 9.**
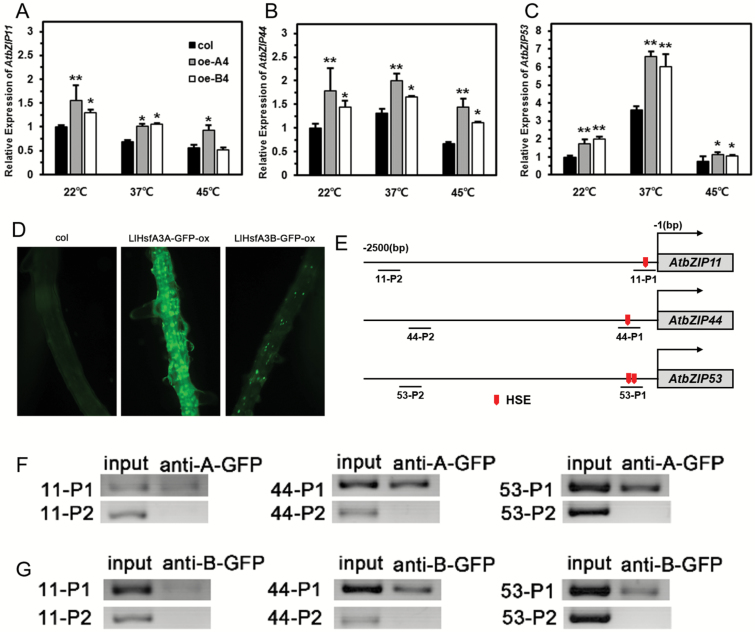
Gene expression analysis of *AtbZIP11*, *AtbZIP44*, and *AtbZIP53*, and examination of binding of heat-stress element (HSE) of LlHsfA3A and LlHsfA3B *in vivo*. (A–C) Expression levels determined by qPCR after 1 h of heat stress (HS) treatment in the wild-type and transgenic lines. Data are means (±SD) of three biological replicates. Significant differences between the wild-type and transgenic plants were determined using Student’s *t*-test (*P<0.05, **P<0.01). (D) GFP fluorescence observed in the roots of the LlHsfA3A-GFP-ox and LlHsfA3B-GFP-ox lines. (E) HSEs located in the promoter; the probe sequences analysed in the ChIP assays are indicated. (F, G) PCR analysis of immunoprecipitated DNA. Different P1-probes containing the HSE sequence were detected in the chromatin DNA immunoprecipitated with the anti-GFP antibody. P2-probes without the HSE sequence served as negative controls (not detected). The input controls and test probes were performed with 33 and 36 cycles, respectively. One representative image of three independent experiments is shown.

Due to the significant induction of *AtproDH1* and *AtproDH2* in both transgenic lines, we examined the expression of their direct upstream regulators, which included eight bZips (basic leucine zipper transcription factors) (Satoh *et al.*, 2004; Weltmeier *et al.*, 2006). Under normal conditions, the expression levels of *AtbZIP1*, *AtbZIP2*, *AtbZIP10*, *AtbZIP25*, and *AtbZIP63* in oe-A4 and oe-B4 were not apparently affected (Supplementary Fig. S11), but *AtbZIP11*, *AtbZIP44*, and *AtbZIP53* were induced; under HS, their expressions in oe-A4 and oe-B4 were still higher than in the wild-type (Fig. 9A–C). By analysing the promoter (–1500 bp) of these eight *bZip* genes, it was found that only *AtbZIP11*, *AtbZIP44*, and *AtbZIP53* contained the conserved HSE (nGAAnnTTCn) (Fig. 9E). To investigate whether they were the target genes of LlHsfA3A and LlHsfA3B, we conducted a ChIP assay, and the interaction of LlHsfA3A or LlHsfA3B with the HSE sequence in these promoters was confirmed (Fig. 9D–G).

### Proline accumulation is decreased in overexpression plants under salt stress

**Fig. 10.**
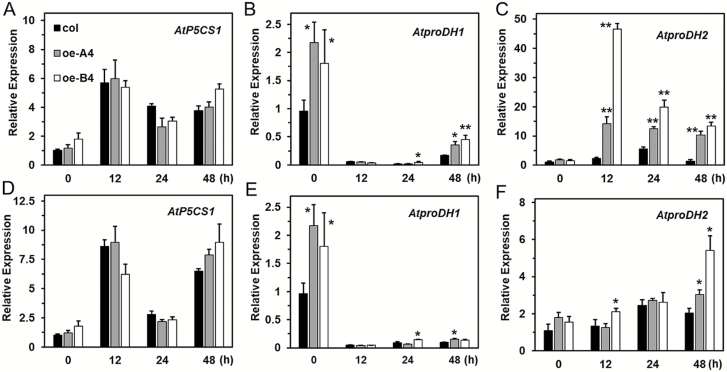
Gene expression analysis of *AtP5CS1*, *AtproDH1*, and *AtproDH2* by qPCR during salt stress. (A–C) Relative expression in the wild-type and transgenic lines after salt treatment in the presence of 2% sucrose, and (D–F) expression in the absence of sucrose. The raw data were normalized by using *AtActin2* as an internal reference. Data are means (±SD) of three biological replicates. Significant differences between the wild-type and transgenic plants were determined using Student’s *t*-test (*P<0.01, **P<0.001).

Under the stress of salt and mannitol, normal proline metabolism was required for establishing tolerance. Therefore, we evaluated the expression of several key genes, namely *AtP5CS1*, *AtproDH1*, and *AtproDH2*, under salt and mannitol stress. With or without sucrose, the expression trend of *AtP5CS1* was similar among the test lines (Fig. 10A, D). *AtproDH1* was strongly repressed by NaCl in all lines; without sucrose, after 48 h, its expression in oe-A4 and oe-B4 was markedly increased compared to the wild-type (Fig. 10B, E). Without sucrose, *AtproDH2* was sharply induced in oe-A4 and oe-B4 during the entire treatment phase (Fig. 10C), but with sucrose, its expression stayed at a low level similar to that of the wild-type after 24 h of treatment, and was weakly up-regulated after 48 h (Fig. 10F). By contrast, even without sucrose, the expression levels of *AtP5CS1*, *AtproDH1*, and *AtproDH2* in oe-A4 and oe-B4 were not much different from that of the wild-type under mannitol stress (Supplementary Fig. S12).

**Fig. 11.**
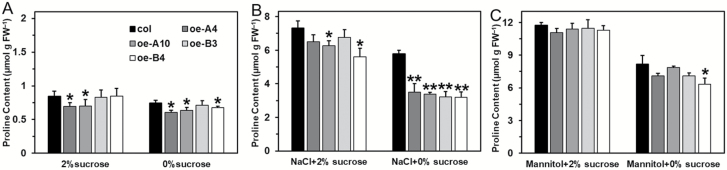
Proline accumulation of wild-type and transgenic plants in response to salt and mannitol stresses. (A) The 7-d-old seedlings were transferred to 1/2-MS medium with or without sucrose and grown for 72 h under normal conditions. (B) The 7-d-old seedlings were transferred to 1/2-MS medium supplemented with NaCl (150 mM) with or without sucrose and grown for 72 h. (C) The 7-d-old seedlings were transferred to 1/2-MS medium supplemented with mannitol (300 mM) with or without sucrose and grown for 72 h. Data are means (±SD) of three independent experiments. Significant differences between the wild-type and transgenic plants were determined using Student’s *t*-test (*P<0.05, **P<0.01).

Under salt stress, more proline accumulated in the wild-type than in the transgenic lines; however, after removing sucrose, proline accumulation was severely inhibited in both the oe-A and oe-B lines (Fig. 11B). When subjected to mannitol stress, the wild-type and transgenic plants accumulated similar levels of proline. Notably, unlike the salt treatment, the proline content of transgenic plants was just slightly lower than that of the wild-type when sucrose was absent (Fig. 11C).

### Transient overexpression of *LlHsfA3A* or *LlHsfA3B* in lily affects proline accumulation and tolerance behaviors under heat and salt stress

**Fig. 12.**
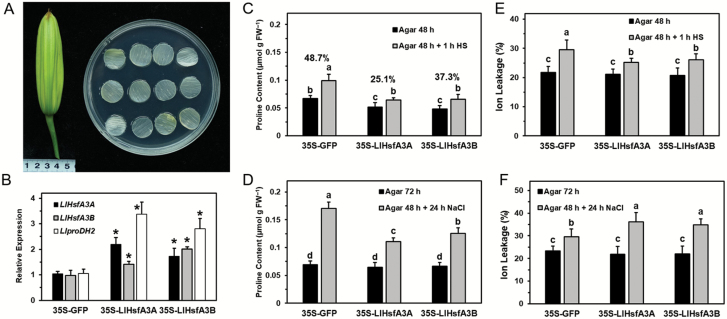
Transient expression of *LlHsfA3A* and *LlHsfA3B* in lily reduces proline accumulation under salt stress. (A) Flower buds and discs taken from the inner petal (see Methods). (B) Expression levels of *LlHsfA3A*, *LlHsfA3B*, and *LlproDH2* in overexpressed petals relative to the control (set as 1) as determined by qPCR. Data are means (±SD) of three independent experiments. Significant differences compared with the control were determined using Student’s *t*-test (*P<0.05). (C, D) Proline content of discs determined after the treatments described in the Methods. (E, F) Relative ion leakage (%) of discs determined after the treatments described in the Methods. Data are means (±SD) of three independent experiments. Each biological replicate included 20 discs. The percentages shown in (C) indicate the within-pair relative increase. Different letters indicate significant differences as determined using Student–Newman–Keuls test (*P*<0.05).

A transient assay with lily petal discs was used to evaluate the roles of *LlHsfA3A* and *LlHsfA3B* under heat and salt stress (Fig. 12A). qPCR analysis showed that the accumulation levels of *LlHsfA3A*, *LlHsfA3B*, and *LlproDH2* (a proline dehydrogenase gene of lily) were enhanced after 48 h of transformation, and the expression of *LlHsfA3A* and *LlHsfA3B* could be stimulated by each other (Fig. 12B). The proline content of *LlHsfA3A*- and *LlHsfA3B*-overexpressing discs was altered after 48 h, but was the same after 72 h; for the treatment at 42 °C for 1 h, at 48 h proline increased in all the test discs, but accumulation in the *LlHsfA3A*- and *LlHsfA3B*-overexpressing discs was lower than the control, and their rates of increase were also lower. Under the 24-h salt treatment, proline accumulation decreased in the *LlHsfA3A*- and *LlHsfA3B*-overexpressing discs when compared with that of the control (Fig. 12C, D). The relative ion leakage of both overexpressing discs was lower than that of the control after HS but higher after salt stress, which suggested better thermotolerance and poorer salt-tolerance with overexpression (Fig. 12E, F).

### Exogenous proline impairs thermotolerance and recovers salt tolerance of transgenic Arabidopsis

When exogenous proline was applied, seed germination of the oe-A and oe-B lines was improved under salt stress, and oe-B3 even recovered to match the wild-type level (Supplementary Fig. S13). This indicated that exogenous proline could greatly ameliorate the salt sensitivity of transgenic lines.

**Fig. 13.**
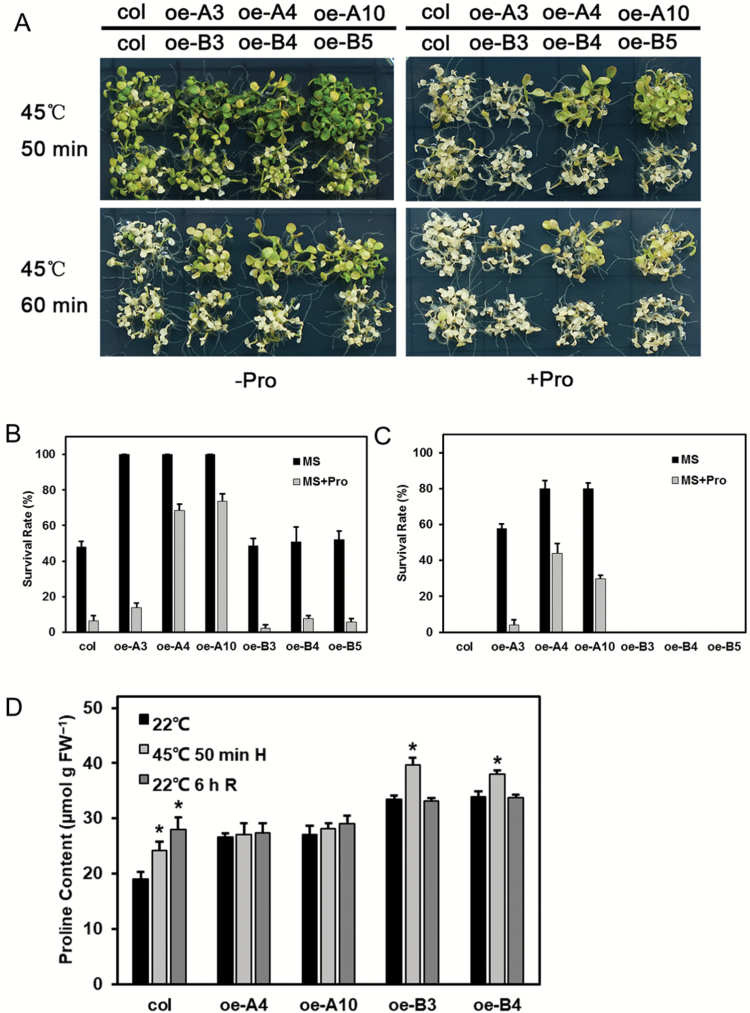
Responses of the transgenic plants to heat stress with exogenous proline (Pro). (A) Seedlings grown on proline-supplemented medium were exposed to heat stress, and images were taken after 7 d. One representative image of three independent experiments is shown (each experiment included over 30 seedlings of each line). (B, C) The survival rate of the test lines was recorded after a 7-d recovery period at 22 °C. Data are means (±SD) of three independent experiments. (D) Proline content of the wild-type and transgenic lines. Data are means (±SD) of three independent experiments. Significant differences compared with the 22 °C control were determined using Student’s *t*-test (*P*<0.05).

Seedlings grown on proline-supplemented MS medium were directly subjected to HS at 45 °C. Although abundant proline impaired the thermotolerance of all lines, the oe-As still showed greater tolerance than did the wild-type and oe-Bs (Fig. 13A–C). Determination of proline content revealed that all lines had accumulated substantial amounts, with increases up to about 30-fold relative to normal growing conditions. Specifically, proline content of the transgenic lines was higher than that of the wild-type, and highest in the oe-B lines. After HS, proline increased in both the wild-type and oe-B lines, but this trend was not apparent in the oe-A lines (Fig. 13D).

## Discussion

Although Hsfs have been increasingly studied in model plants in recent decades, they remain poorly understood in other plant species. Almost all reports to date have implicated class-A Hsfs as playing a positive role in different abiotic stresses (von Koskull-Döring *et al.*, 2007; Scharf *et al.*, 2012); few studies, however, have considered their negative effects on plant responses (Li *et al.*, 2013). In this work, we identified two HsfA3s from lily that function as activators in the HSR, but that negatively regulate the salt response. The results of this study suggest that the proline-mediated resistance pathway affected by them is a potential reason for the differential plant responses to heat and salt stress.

### Two functional *HsfA3*s from lily, *LlHsfA3A* and *LlHsfA3B*, are differentially involved in thermotolerance

Tomato and Arabidopsis each have only one HsfA3, SlHsfA3 and AtHsfA3, respectively; under room temperature conditions, they are distributed in the cytoplasm and nucleus, but after HS some of them migrate to the nucleus (Nover *et al.*, 2001; Guo *et al.*, 2008; Yang *et al.*, 2016). Hence, SlHsfA3 and AtHsfA3 are nuclear-cytoplasm proteins that must enter the nucleus to fulfil their functions during HS (Bharti *et al.*, 2000; Yoshida *et al.*, 2008; Li *et al.*, 2013). In contrast, two HsfA3s could be found in different cultivars of lily (Fig. 1), and they showed different localizations: LlHsfA3A was localized in the cytoplasm and the nucleus, much like SlHsfA3 and AtHsfA3, whereas LlHsfA3B was restricted to the nucleus (Fig. 2). Different protein localizations may point to their different functions. In Arabidopsis, overexpression of *AtHsfA3* can improve its BT and AT (Yoshida *et al.*, 2008; Li *et al.*, 2013; Hu *et al.*, 2015). Similarly, the overexpression of *LlHsfA3A* improved the BT and AT of transgenic Arabidopsis; however, *LlHsfA3B* overexpression had no apparent effect on BT, only improving AT (Fig. 4). Nevertheless, transient overexpression of either *LlHsfA3A* or *LlHsfA3B* in lily was able to improve its BT, which may be due to LlHsfA3B activating the expression of *LlHsfA3A* in lily (Fig. 12). Given the complexity of the lily HsfA3 group having at least two members, the regulating network of LlHsfA3 may differ considerably from model plants.

### Proline accumulation is decreased under salt stress, with overexpression of *LlHsfA3A* or *LlHsfA3B* contributing to salt sensitivity

Proline accumulation occurs in a variety of plant species in response to environmental stresses, such as drought, salinity, extreme temperatures, UV radiation, and heavy metals (Siripornadulsil *et al.*, 2002; Ashraf and Foolad, 2007; Verbruggen and Hermans, 2008; Szabados and Savouré, 2010; Ben Rejeb *et al.*, 2015). Much experimental evidence suggests that there is a positive correlation between proline accumulation and stress tolerance, in particular in relation to salt, osmotic, and dehydration stress tolerances (Delauney and Verma, 1993; Chiang and Dandekar, 1995; Verslues and Bray, 2006; Sharma and Verslues, 2010; Estrada-Melo *et al.*, 2015). In our study, overexpression of *LlHsfA3A* or *LlHsfA3B* caused salt sensitivity (Fig. 5), and the salt-sensitive phenotype was aggravated by low sucrose (Fig. 6). Further analysis revealed that both LlHsfA3A and LlHsfA3B elevated the expression of *AtbZIP11*, *AtbZIP44*, and *AtbZIP53* (Fig. 9), which may contribute to proline catabolism through the activation of *AtproDH1* and *AtproDH2* (Satoh *et al.*, 2004; Weltmeier *et al.*, 2006; Hanson *et al.*, 2008; Dietrich *et al.*, 2011). This activation may have caused the proline accumulation to decrease under salt stress (Fig. 11) and caused the plants to exhibit the concurrent salt sensitivity. Why the lack of sucrose exacerbated the salt sensitivity of the overexpression lines may be due to three reasons. (1) Sucrose is an organic osmolyte, and when absorbed by plants it may participate in osmotic regulation under salt stress (Khoyi and Hesari, 2007; Cui *et al.*, 2010). (2) Under salt stress, high sucrose levels inhibit the expression of *AtproDH2* (Funck *et al.*, 2010), which may be attributable to the post-transcriptional translation of *AtbZIP11*, *AtbZIP44*, and *AtbZIP53* being repressed by sucrose (Wiese *et al.*, 2004; Hanson *et al.*, 2008). (3) Sucrose can also be used as an energy source, and it plays a role in energy conversion; it has been demonstrated that AtbZIP11 interacts with AtbZIP63 as a key regulator of the starvation response, thereby affecting target gene expression and ultimately primary metabolism (Mair *et al.*, 2015). Recently, Li *et al.* (2013) reported that tomato *SlHsfA3*, when overexpressed in Arabidopsis, produced a salt-sensitive phenotype at the germination stage, but this disappeared in later stages of plant growth. This inconsistency with our results may simply reflect the differing treatments applied in each study; at the post-germination stage, when sucrose was removed from the medium, the oe-A and oe-B lines still exhibited salt sensitivity. Furthermore, mannitol sensitivity of the transgenic plants was not observed in our study (Fig. 7). We speculate that mannitol stress could have masked the effects of LlHsfA3A and LlHsfA3B on proline accumulation (Fig. 11).

### Appropriate proline catabolism under HS may benefit thermotolerance

The positive regulatory role of proline accumulation under salt and osmotic stress is precise, but its physiological function under HS remains controversial (Rizhsky *et al.*, 2004; Miller *et al.*, 2009; Verslues and Sharma, 2010; Cabassa-Hourton *et al.*, 2016). Some reports have shown proline accumulating during HS; for example, when subjected to 41 °C, the proline content of the first leaf of barley (*Hordeum vulgare*) and radish (*Raphanus sativus*) showed a slight increase (Chu *et al.*, 1974). However, in other studies, proline accumulation seems not to have occurred under HS (Yoshiba *et al.*, 1995; Hua *et al.*, 2001). In Arabidopsis, more in-depth studies have shown that proline accumulation is not beneficial during HS, but that proline catabolism and the proline/P5C cycle do play crucial roles in the plant’s response to high temperatures (Larkindale and Vierling, 2008; Miller *et al.*, 2009). Lv *et al.* (2011) reported that proline overaccumulation during HS resulted in the thermo-sensitivity of Arabidopsis, and explained this phenotype as being driven by abundant proline activating the proline/P5C cycle to oxidize proline to produce excess ROS. The *p5cdh* mutant has been shown as more sensitive to HS, and due to its inability to completely oxidize proline the intermediate P5C is transported to the cytosol and reduced to proline, which is then transported back into the mitochondria where it enters the proline/P5C cycle to generate ROS (Miller *et al.*, 2009). ROS is generated from proline oxidation, which may be harmful for thermotolerance, but these results also indicate that proline oxidation is not prohibited under HS. AtproDH1 is the rate-limiting enzyme for catalysing proline degradation, and its mutant can block proline oxidation but still show thermo-sensitivity. In addition, the expression of *AtproDH1* was shown to be activated by the acclimated heat treatment (37 °C) to inhibit proline increase during HS (Larkindale and Vierling, 2008). Together, those findings suggest the ability to degrade proline via proDH is essential for thermotolerance, and the activation of proline oxidation is an adaptive response to mitigate subsequent lethal HS.

In this study, the elevated *AtproDH* expression could be interpreted as a similar adaptive reaction without risking the generation of excess ROS, because *AtP5CDH* was also up-regulated and no excess proline accumulated during HS in the tolerant lines (Fig. 8). In a previous study, the activated proline oxidation was found to proactively stimulate the ROS-scavenging system (Ben Rejeb *et al.*, 2014), which should assist plants in adapting to HS. In addition, our preliminary results with *AtproDH1* transgenic plants also showed evidence of increased thermotolerance in them (unpublished data). Therefore, the mildly accelerated proline catabolism in the transgenic lines may have contributed to the enhanced thermotolerance. We also observed that excess proline was harmful to the thermotolerance of all tested lines, but the oe-A lines still showed greater tolerance than did the other lines after the BT treatment (Fig. 13). This suggests that thermotolerance is not only related to the absolute proline level but that it is also closely related to proline metabolic changes occurring under HS. During transient overexpression of *LlHsfA3A* or *LlHsfA3B* in the lily petal discs it was observed that *LlproDH2* was induced and proline accumulation was decreased (Fig. 12), which may have also contributed to the enhanced thermotolerance of these discs. Although overexpression in Arabidopsis activated proline oxidation, the proline content of transgenic lines was not always lower than that of the wild-type (Fig. 8). This may be due to the plants being able to adjust the proline cycle to establish homeostasis for normal growth and development under normal conditions, with homeostasis being specific to each individual (Verslues and Sharma, 2010; Kavi Kishor and Sreenivasulu, 2014). This plasticity may also help explain the different proline accumulations observed between the 48- and 72-h transient assays in the petal discs (Fig. 12). Gene ectopic-overexpression not only affects total proline, but also probably disturbs the required tissue-specific proline synthesis and catabolism, which may be an important reason for the fact that the tolerant and sensitive phenotypes of the transgenic lines did not closely depend on the transcript levels of *LlHsfA3A* and *LlHsfA3B* in this study (Figs 4, 5). The proper co-ordination of proline metabolism in different tissues of Arabidopsis has been confirmed as being crucial for enacting tolerance (Verbruggen and Hermans, 2008).

In conclusion, our results show that LlHsfA3A and LlHsfA3B are implicated in proline-mediated regulation of stress responses. Both can activate the process of proline catabolism in overexpressing plants, which may play a potential role in the differing tolerance behaviors observed under heat and salt stress.

## Supplementary data

Supplementary data are available at *JXB* online.

Table S1. Primers used for RT-PCR detection in the different lily cultivars.

Table S2. Primers used for plasmid reconstruction.

Table S3. Primers used for qPCR.

Table S4. Primers used for the identification of mutant and transgenic plants.

Table S5. Primers used for the chromatin immunoprecipitation assay.

Fig. S1. Sequence analysis of LlHsfA3A and LlHsfA3B.

Fig. S2. Histochemical analysis of *LlHsfA3A* and *LlHsfA3B* promoter-GUS transgenic plants after treatments.

Fig. S3. Molecular analysis of the *LlHsfA3A* and *LlHsfA3B* transgenic Arabidopsis lines.

Fig. S4. The identified T-DNA insertion *hsfa3* mutant and the complemented mutant determined by overexpression of *LlHsfA3A* and *LlHsfA3B*.

Fig. S5. Relative expression levels of some target genes of AtHsfA3 in the transgenic plants.

Fig. S6. Seed germination of mutant and complemented lines in response to salt stress.

Fig. S7. Root growth of wild-type and transgenic plants on 1/2 MS medium with different sucrose concentrations.

Fig. S8. Root elongation and seedling growth of transgenic plants in response to mannitol stress and different levels of sucrose.

Fig. S9. Determination of proline content of transgenic and wild-type plants at different developmental stages.

Fig. S10. qPCR analysis of expression of genes involved in proline metabolism.

Fig. S11. qPCR analysis of *AtbZIP1*, *AtbZIP2*, *AtbZIP10*, *AtbZIP25*, and *AtbZIP63* gene expression under normal conditions.

Fig. S12. qPCR analysis of *AtP5CS1*, *AtproDH1*, and *AtproDH2* gene expression under mannitol stress.

Fig. S13. Responses of the transgenic plants to salt stress with exogenous proline.

Supplementary Tables and Figures
