# Supplementary material for: Overexpression of lily HsfA3s in Arabidopsis confers increased thermotolerance and salt sensitivity via alterations in proline catabolism
Source: J Exp Bot. 2018 Jan 31;69(8):2005–21. doi: 10.1093/jxb/ery035 (PMC6018920; doi:10.1093/jxb/ery035)
Supplement: Supplementary Tables and Figures [file ery035_suppl_supplementary_tables_and_figures.pdf]

Overexpression of two novel *HsfA3s* from lily in Arabidopsis confer increased thermotolerance and salt sensitivity via alterations in proline catabolism

Ze Wu[1], Jiahui Liang[1], Chengpeng Wang[1], Xin Zhao[1], Xionghui Zhong[2], Xing Cao[3], Guoqing Li[1], Junna He[1*], Mingfang Yi[1*]

[1] Beijing Key Laboratory of Development and Quality Control of Ornamental Crops, College of Horticulture, China Agricultural University, Beijing 100193, China

[2] State Key Laboratory for Biology of Plant Diseases and Insect Pests, Institute of Plant Protection, Chinese Academy of Agricultural Sciences, Beijing 100193, China

[3] College of Agronomy, Liaocheng University, Liaocheng 252059, China

* Corresponding authors:

Mingfang Yi

China Agricultural University, 2 Yuanmingyuan West Road, Haidian district, Beijing 100193, China

Email: ymfang@cau.edu.cn

Junna He

China Agricultural University, 2 Yuanmingyuan West Road, Haidian district, Beijing 100193, China

Email: hejunna@cau.edu.cn

Supplemental Information Table S1. Primers used for RT-PCR detection in the different lily cultivars.

18S rRNA	5’-AGTTGGTGGAGCGATTTGTCT-3’<br>5’-CCTGTTATTGCCTCAAACTTCC-3’
LlHsfA3A	5’-ACGACATGAAGCGGAAGCAACTCT-3’<br>5’-TCTCTCTTGAGAATCATCACTGTCCA-3’
LlHsfA3B	5’-GTCAGCAGCAGCACAGTCATGGCGA-3’<br>5’-TGTGGTTCTCCGAACAACTGGTCATGA-3’

Supplemental Information Table S2. Primers used for plasmid reconstruction.

pCAMBIA1300	*p1300-LlHsfA3A*	5’-ACGCGTCGACATGGAAAT CCAAAATCTCCAATTCGA -3’<br>5’- CGGGGTACCCTATGGATT CAAATCTTTTGGAAGATCTTCATTC -3’
pCAMBIA1300	*p1300-LlHsfA3B*	5’-ACGCGTCGACATGGAAATCC AAAACCTCCAATTCGATCG-3’<br>5’-CGGGGTACCTCAGTGGCTCT TCAGCCCGGCTATG-3’
p1300-GFP-C	*p1300-GFP-C-LlHsfA3A*	5’-ACGCGTCGACATGGAAAT CCAAAATCTCCAATTCGA -3’<br>5’- CGGGGTACCTGGATT CAAATCTTTTGGAAGATCTTCATTC -3’
p1300-GFP-C	*p1300-GFP-C-LlHsfA3B*	5’-ACGCGTCGACATGGAAATCC AAAACCTCCAATTCGATCG-3’<br>5’-CGGGGTACCGTGGCTCT TCAGCCCGGCTATG-3’
p1300-GFP-N	*p1300-GFP-N-LlHsfA3A*	5’-ACGCGTCGACATGGAAAT CCAAAATCTCCAATTCGA -3’<br>5’- CGGGGTACCTGGATT CAAATCTTTTGGAAGATCTTCATTC -3’
p1300-GFP-N	*p1300-GFP-N-LlHsfA3B*	5’-ACGCGTCGACATGGAAATCC AAAACCTCCAATTCGATCG-3’<br>5’-CGGGGTACCGTGGCTCT TCAGCCCGGCTATG-3’
pCAMBIA1391	*p1391-LlHsfA3A*	5’-AACTGCAGGAAGTTCGTAGTAC AAATATTTCCTCTGTTCCC-3’<br>5’-TCCCCCGGGTGGTGGATTTGAGG GGGAAAGAGAAGGAGTG-3’
pCAMBIA1391	*p1391-LlHsfA3B*	5’-AACTGCAGCTCTATGCTTGCAGA TCCCTTAACCAAGG-3’<br>5’-TCCCCCGGGTGGGTGGCGTGGG AGAAGTGAATTG-3’
pGBKT7	*pBD-*	5’-CGGAATTCATGGTTTCCAGTTCT

	LlHsfA3A	CCTCCTCCCCCGCCG-3’<br>5’-ACGCGTCGACCTATGGATTCAAATCT<br>TTTGGAAGATCTTCATTC-3’
pGBKT7	*pBD-LlHsfA3m*	5’-CGGAATTCATGGTTTCCAGTTCT<br>CCTCCTCCCCCGCCG-3’<br>5’- ACGCGTCGACCTATGGATTCAAATCT<br>TTTGGAAGATCTTCATTC -3’
pGBKT7	*pBD-LlHsfA3d*	5’-CGGAATTCATGGTTTCCAGTTCT<br>CCTCCTCCCCCGCCG-3’<br>5’- ACGCGTCGACCTAGCTATGGATTCAAA<br>TCTTTTGGAAGATCTTCA -3’
pGBKT7	*pBD-LlHsfA3B*	5’-CGGAATTCATGGTTTCCGGTTCT<br>CCGCTGCCTCCGCCG-3’<br>5’-ACGCGTCGACTCAGTCAGTGGCTCTTCA<br>GCCCGGCTATG-3’
pGBKT7	*pBD-LlHsfA3Bm*	5’-CGGAATTCATGGTTTCCGGTTCT<br>CCGCTGCCTCCGCCG-3’<br>5’-ACGCGTCGACTCAGTCAGTGGCTCTTCA<br>GCCCGGCTATG-3’
pGBKT7	*pBD-LlHsfA3Bd*	5’-CGGAATTCATGGTTTCCGGTTCT<br>CCGCTGCCTCCGCCG-3’<br>5’-ACGCGTCGACTCAGTCAGTGGCT<br>CTTCAGCCCGGCTATGGG -3’

Supplemental Information Table S3. Primers used for qPCR.

LlHsfA3A	Using for qPCR assay of HS leaves	5’-CTTGGTTTAAGTACGCCAGTGGAAG-3’<br>5’-GTAAAATATTGTAAAAGAACATGAAGCCTATGG-3’
LlHsfA3B	Using for qPCR assay of HS leaves	5’-TGAAGAGCCACTGAGCACAAGTC-3’<br>5’-CAGTTATGATGATCTGTAGTCCTTTGTC-3’
LlHsfA3A	Using for qPCR assay of the transient-overexpressed petal discs	5’-CAGTTCACCTTATCCGCTGCGA-3’<br>5’-CTTAAAGTTCAGATGGTCGTGTCCTTG-3’
LlHsfA3B	Using for qPCR assay of the transient-overexpressed petal discs	5’-TAGGGATTGATGCTGGAGCTGGTTC-3’<br>5’-GCTATGGGCCACACCTGTCTTG-3’
LlproDH2	Using for qPCR assay of the	5’-AGCAGGTGATGCCCTACCTCCTGAGA-3’<br>5’-TCACTGTTATCTTGAACTCCTCCGGAGA-3’

	transient-overexpressed petal discs	
18S rRNA		5’-AGTTGGTGGAGCGATTTGTCT-3’<br>5’-CCTGTTATTGCCTCAAACTTCC-3’
AtP5CS1	*At2g39800*	5’-GCAAAGTTGGACTATCCAGCAG-3’<br>5’-CTTGGTCCACCATACAAAGTGAC-3’
AtP5CS2	*At3g55610*	5’-GATGCTGAGGATGAGGGTTATT-3’<br>5’-GAGCGGCTAAGCTGTCATTA-3’
AtP5CR	*At5g14800*	5’-TTACCCCGAGAGCTTGCATTGAGT-3’<br>5’-GCCCGGAAAGAGCCTTTCTCTAGT-3’
AtOAT	*At5g46180*	5’-TCCCGACGGTTACTTGAAAGC-3’<br>5’-CAGGACGAATTTCTTCCCAATCAC-3’
AtproDH1	*At3g30775*	5’-CTCGCAACACATAACGCTGATTCG-3’<br>5’-GCCATCATTCCCCGGTTCTCATAA-3’
AtproDH2	*At5g38710*	5’-CGTCGAAGCTGCTAAAACCCT-3’<br>5’-CGTTCGATTCTTGACATCTAAG-3’
AtP5CDH	*At5g62530*	5’-GAACCCACGGATGACCCTCTTC-3’<br>5’-GCCATGCAACATAATCAACCTCCTG-3’
AtbZIP1	*At5g49450*	5’-TCAGCGTTAAACTCGTCGTAGCAA-3’<br>5’-AACGCGGGTCTTAGATCGGAGAAG-3’
AtbZIP2	*At2g18160*	5’-TCACCGCTCAGATGGAGGAGCTT-3’<br>5’-TCCTGCACCGTTGGATTGAACAAG-3’
AtbZIP10	*At4g02640*	5’-TTTTTCGGCCATGCTGAATCGTTC-3’<br>5’-TTACTCCAAGCGCCAACCCGTA-3’
AtbZIP11	*At4g34590*	5’-GGCATGTGTTCGAACCCTCTGGT-3’<br>5’-AGACGCCATGAGAGGCTGGT-3’
AtbZIP25	*At3g54620*	5’-AGGAGGATGCTCTCAAACCGAGAA-3’<br>5’-CGGCTCTTAATTGGCCTACCTGTG-3’
AtbZIP44	*At1g75390*	5’-TTCGACGGCGTGATGAATCCTATG-3’<br>5’-CAGCAGTAGAAGCAGAAGCCATGA-3’
AtbZIP53	*At3g62420*	5’-TGGGGTCGTTGCAAATGCAAACAA-3’<br>5’-CCGTGGCGTACCTCGGATCATTAT-3’
AtbZIP63	*At5g28770*	5’-TCAGAACAAGCCTCTCTTGCT-3’<br>5’-CACCAGAGAGCTCAGATCCA-3’
AtGolS1	*At2g47180*	5’-AGCCGTTCATCACCGCTCTTAC-3’<br>5’-ACTCCTGGCAACATTCAAGCAG-3’
AtGolS2	*At1g56600*	5’-AAGAAGCAACAGACACTTCAGCAG-3’<br>5’-TGAAGAGGCGTATGCAGCAAC-3’
AtGolS4	*At1g60470*	5’-AGATGCGGAAGAAACCGTTAC-3’<br>5’-CAGCAGAAGGAGCAGGAAAGTAG-3’
AtHsp22.0	*At4g10250*	5’-ACTACTCCAGGCAGCTTGCTA-3’<br>5’-CTTGAATGGATCAGGGAACC-3’
AtHsp25.3	*At4g27670*	5’-GATCAAGATGCGTTTCGACAT-3’

		5’-TTCTACAGAGATTTTGACGTCTTCTT-3’
AtHsp19.9	*At1g52560*	5’-GAGAAGAAATCTCCTCGACAGAA-3’<br>5’-CCTATAGTCGGAGGAAAGAACTCA-3’
AtHsp70b	*At1g16030*	5’-TGCACGATGTTGTTCTGGTT-3’<br>5’-GCAAAAGCTGTTGAATTTTCG-3’
AtACTIN2	*At3g18780*	5’-TCCCTCAGCACATTCCAGCAGAT-3’<br>5’-AACGATTCCTGGACCTGCCTCATC-3’

Supplemental Information Table S4. Primers used for the identification of mutant and transgenic plants.

AtHsfA3	*At5g03720*<br>*SALK_011131*	5’-GATGCTGTTTCTAAACCAACTCCAATTTCA-3’<br>5’-TCAATGCACGCCGCTCTTTCCTCA-3’
LlHsfA3A		5’-CAGTTCACCTTATCCGCTGCGA-3’<br>5’-CTTAAAGTTCAGATGGTCGTGTCCTTG-3’
LlHsfA3B		5’-TAGGGATTGATGCTGGAGCTGGTTC-3’<br>5’-GCTATGGGCCACACCTGTCTTG-3’

Supplemental Information Table S5. Primers used for the chromatin immunoprecipitation assay.

AtbZIP11	11-P1	5’-CTTTGATTAATGTAGACACTCTAGAAAAC-3’<br>5’-ATCAAAAAGCGTTTTGAGTGTTTGTGCA-3’
	11-P2	5’-AATAAATGGACACACATGTACTTTCTCAG-3’<br>5’-GTCAAAATTTTCTTCGGATCTTGAAATCT-3’
AtbZIP44	44-P1	5’-ACTGTAATATTGTTGAAAGCCATCATTTGA-3’<br>5’-ATGTGTATGGGTTTAATTGTAAATTAATGA-3’
	44-P2	5’-AACTAGTTTTATTGATAATTTATTTGACA-3’<br>5’-TATTGCTTTCTACTAAGGCATATCACATC-3’
AtbZIP53	53-P1	5’-GTGTGTTAGACGTTGCTTTCGTGAGGA-3’<br>5’-AGCTGTGACGCCGCCAGCTGGA-3’
	53-P2	5’-CTGTCAATGATTGTTTACGCAATGTGTTG-3’<br>5’-GAACTGTATCAAGAAGGAGAGTCTTAAGAG-3’

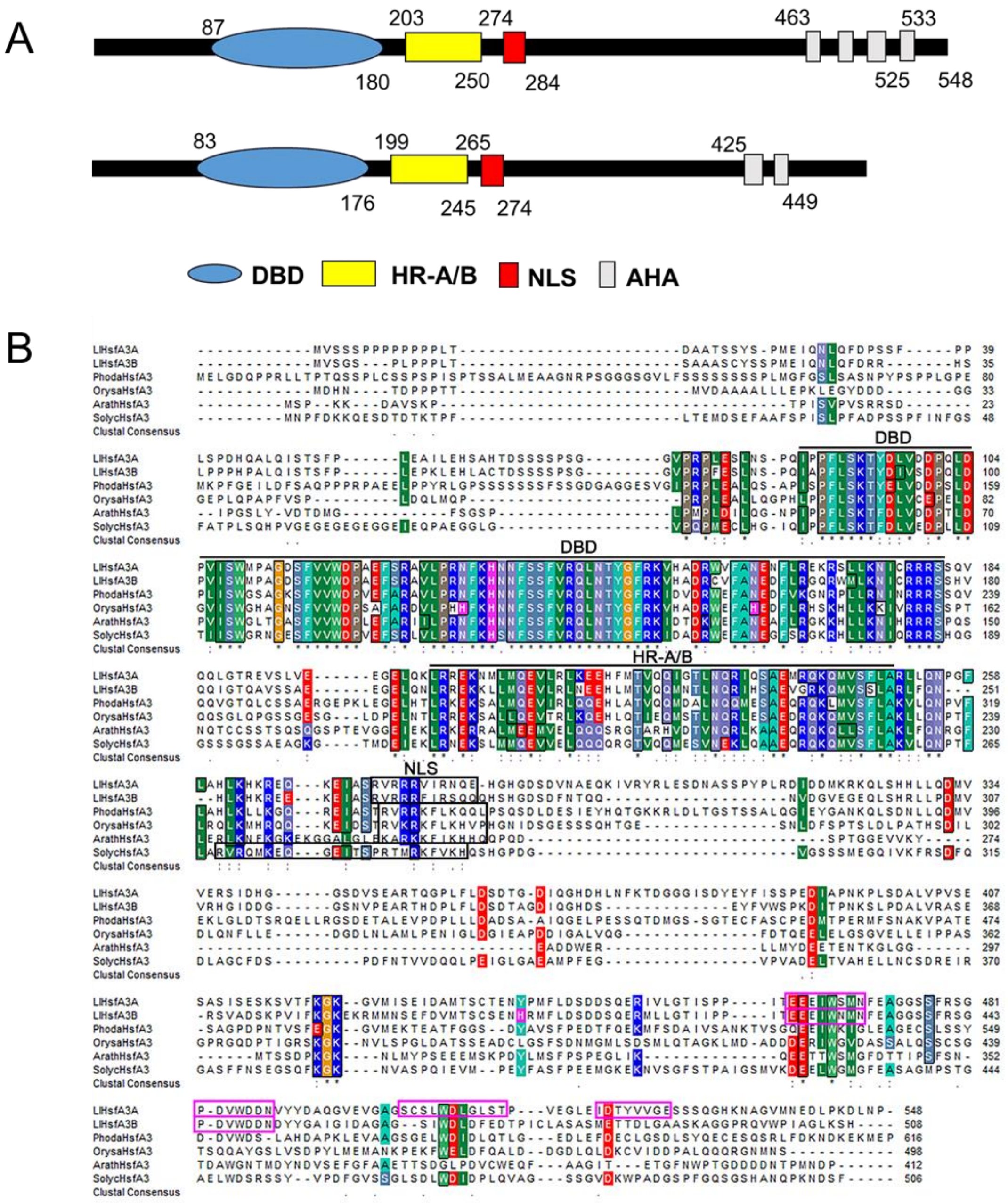

Fig. S1. Sequence analysis of LlHsfA3A and LlHsfA3B proteins.

(A) Modular structures of LlHsfA3A and LlHsfA3B according to the analysis of Heatster online tool (http://www.cibiv.at/services/hsf/). The block diagrams represented their conserved functional domains. DBD (blue), N-terminal DNA binding domain; HR-A/B (yellow), a heptad repeat region of hydrophobic amino acid residues, oligomerization domain (OD); NLS (red), nuclear localization signal; AHA (gray), activator motif. (B) Alignment of the deduced amino acid sequences of LlHsfA3A and

LlHsfA3B with HsfA3 proteins from other plants. The sequences of HsfA3 of *Arabidopsis thaliana* (ArathHsfA3), *Oryza sativa* (OrysaHsfA3), and *Solanum lycopersicum* (SolycHsfA3) acquired from Heatster database. In addition, the sequence of HsfA3 of *Phoenix dactylifera* (PhodaHsfA3) downloaded from GenBank, the Gene ID is LOC103695539. Completely and partly conserved amino acids in proteins were stained, respectively. The conserved domain of DBD and HR-A/B was labeled with stable lines. NLS was labeled with black boxes. The pink boxes indicated the predicted AHA motifs of LlHsfA3A and LlHsfA3B.

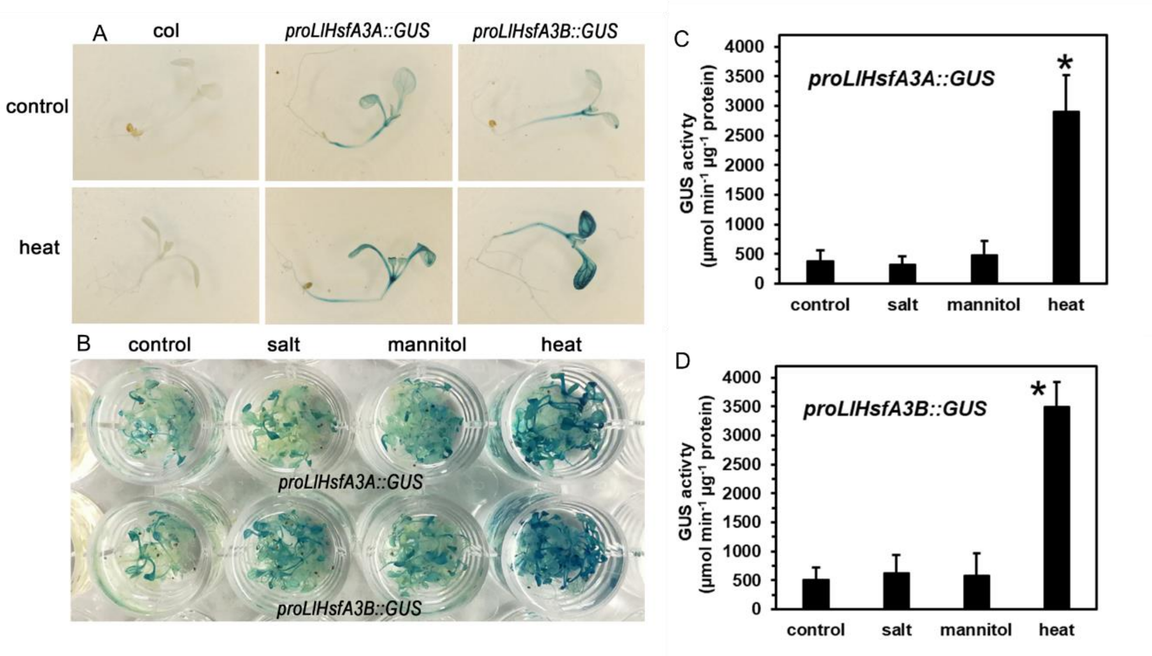

Fig. S2. GUS activity analysis of *LlHsfA3A* and *LlHsfA3B* promoter-GUS transgenic plants after treatments. (A) Histochemical analysis of GUS activity of 7-d-old wild-type and transgenic seedlings grew under normal conditions and treated with 37℃ for 3 h. (B) Seedlings were treated with water (control), salt solution (NaCl, 150 mM) or mannitol solution (300 mM) for 12 h and HS (37℃) for 3 h before being subjected to GUS analysis. (C) Measurement of GUS activity after treatments which described in (B). Data are shown as means ± SD of one representative experiment with three technological repeats. * Significant at $P < 0.05$ compared with the control (Student's *t*-test).

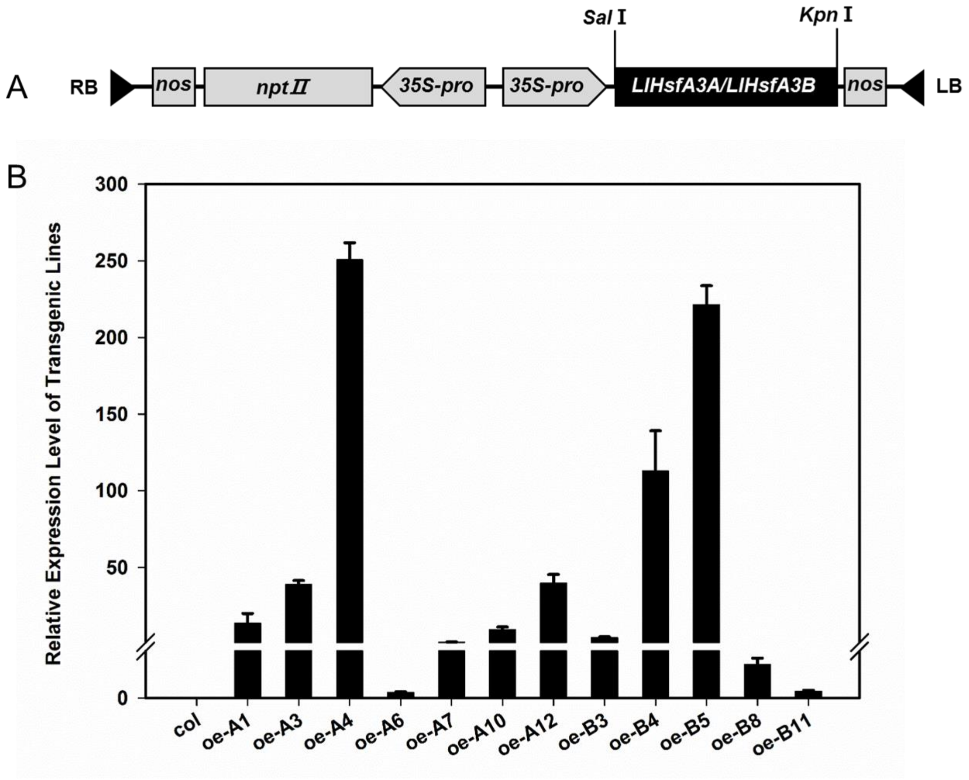

Fig. S3. Molecular analysis of the *LlHsfA3A* and *LlHsfA3B* transgenic Arabidopsis lines. (A) Schematic diagram of the chimeric gene expression construct. The *LlHsfA3A* or *LlHsfA3B* (coding sequence only) are cloned into this vector with *Sal*I and *Kpn*I sites, the vector contained a homomycin resistance gene, *nptII*, they are both under the control of the 35S promoter. RB, right border; LB, left border. (B) 5-d-old seedlings were used to analysis of the expression of the *LlHsfA3A* and *LlHsfA3B* of wild-type and transgenic plants by qPCR. *AtActin2* was used as the endogenous control. The oe-A and oe-B represented LlHsfA3A and LlHsfA3B transgenic lines, respectively, and the number was the code. Data are means ± SD of three biological replicates.

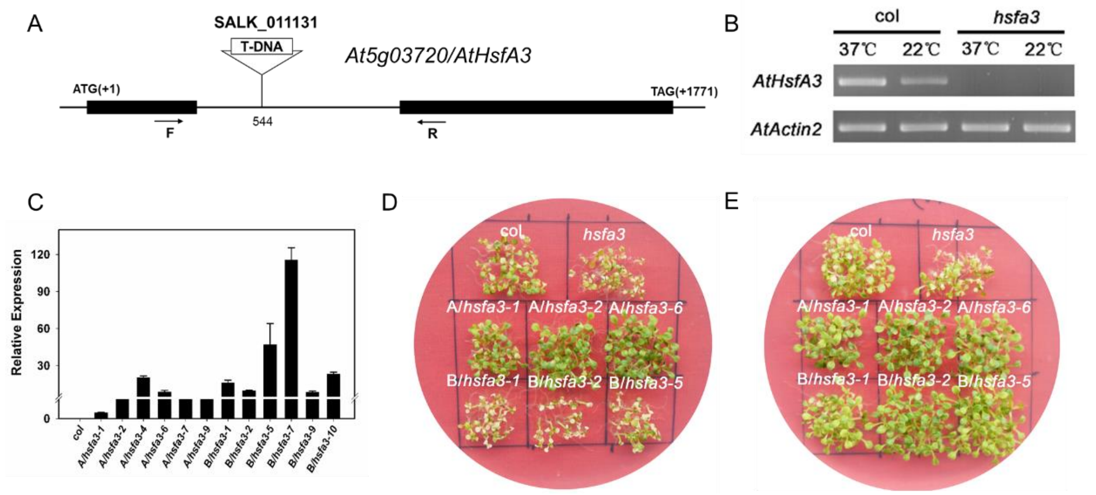

Fig. S4. Identified T-DNA insertion *hsfa3* mutant and the complemented mutant by overexpression of *LlHsfA3A* and *LlHsfA3B*, respectively. (A) Position of SALK_011131 T-DNA insertion in the *At5g03720* (*AtHsfA3*) gene and primers used for RT-PCR. (B) RT-PCR analysis of *AtHsfA3* transcription in wild-type and *hsfa3* mutant seedlings treated with HS 37°C for 1 h. (C) 5-day-old seedlings was used to detect the expression of *LlHsfA3A* and *LlHsfA3B* of wild-type and complemented lines by qPCR. *AtActin2* was used as the endogenous control. *A/hsfAa3* and *B/hsfa3* represented LlHsfA3A and LlHsfA3B complemented lines, respectively, and the numbers behind *A/hsfAa3* or *B/hsfa3* were the code. Data are means ± SD of three replicates. (D) Wild-type, *hsfa3* mutant, and complemented lines were treated with 45°C for 50 min, after 7d, photographed the picture. (E) Wild-type, *hsfa3* mutant, and complemented lines were treated with 37°C for 60 min, following by recovery 2 h at 22°C, then treated with 45°C for 80 min, after 7 days, photographed the picture. One representative from three independent experiments (each treatment included over 30 seedlings of each line) is shown in D, E.

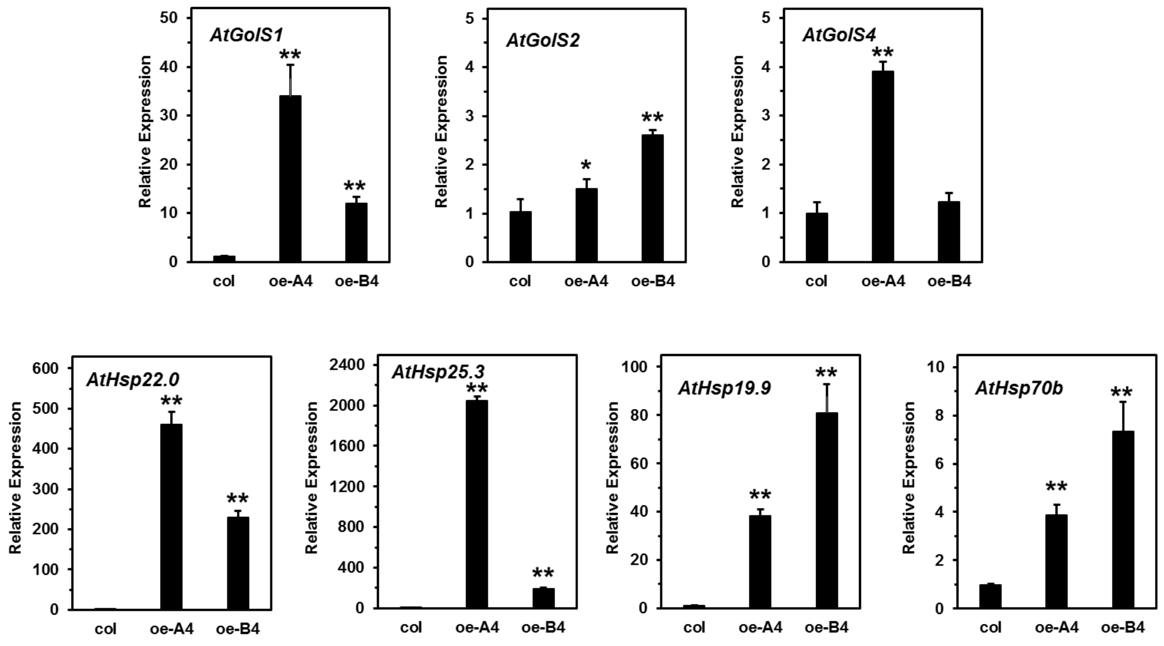

Fig. S5. Relative expression levels of some target genes of AtHsfA3 in the transgenic plants. The detected genes included *AtGolS1*, *AtGolS2*, *AtGolS4*, *AtHsp22.0*, *AtHsp25.3*, *AtHsp19.9* and *AtHsp70b*. The raw data were normalized using *AtActin2* as an internal reference. The data represent means ± SD of three independent experiments. Significant differences between wild-type and transgenic plants are indicated (* $P < 0.05$ and ** $P < 0.01$, Student's *t*-test).

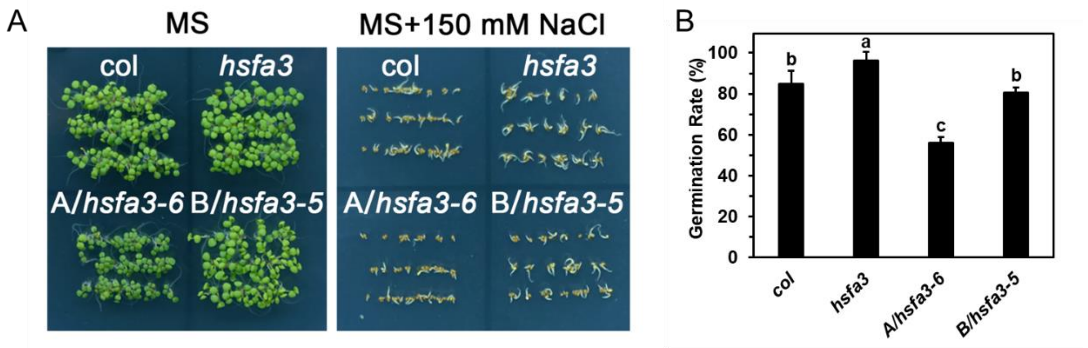

Fig. S6. Seed germination of mutant and complemented lines in response to salt stress. (A) Seed germination on NaCl-treated MS plates photographed after 5 days under light. Mutant line SALK_011131, LlHsfA3A complemented line A/*hsfAa3-6* and LlHsfA3B complemented line B/*hsfAa3-5* were used in this experiment. One representative from three independent experiments (each treatment included over 50 seeds of each line) is shown. (B) 5-day germination of seeds treated with 150 mM NaCl. Bars are means ± SD of three independent experiments. Different letters indicate significant differences among these lines (Student–Newman–Keuls test, $P < 0.05$).

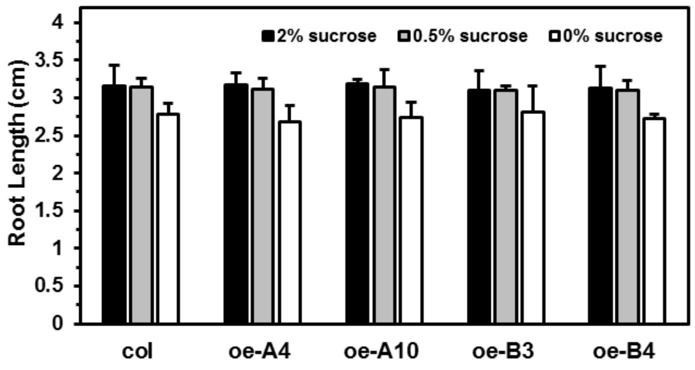

Fig. S7. Root growth of wild-type and transgenic plants on the 1/2 MS medium with different sucrose concentrations. 6-day-old seedlings were transferred in 1/2 MS medium containing 2%, 0.5% and 0% sucrose. The root elongation of each plant was calculated, and the average value of each line with three independent experiments (each experiment included twelve plants of each line). Bars are means ± SD of three independent experiments.

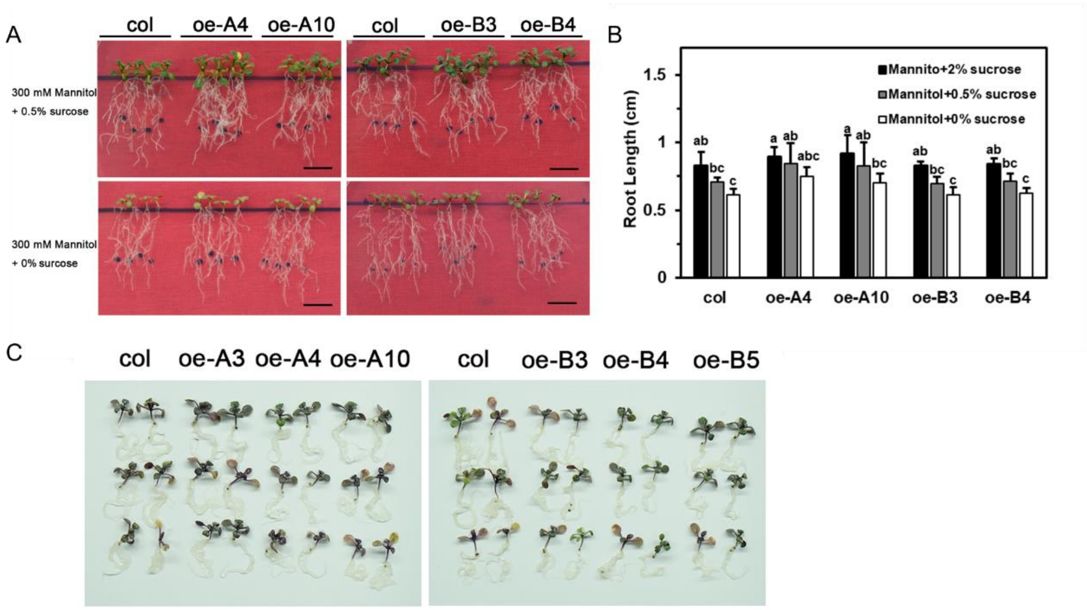

Fig. S8. Root elongation and seedling growth of transgenic plants in response to mannitol stress lacking sucrose. (A-B) 6-day-old seedlings were transferred in 1/2 mannitol-supplemented MS medium containing 2%, 0.5% and 0% sucrose. The root elongation of each plant was calculated, and the average value of each line with three independent experiments (each experiment included twelve plants of each line). Image was taken after 7 days. One representative image from three independent experiments is shown. Bars are means ± SD of three independent experiments. Letters indicate significant differences among different treatments (Student–Newman–Keuls test at P <0.05). (C) 7-day-old seedlings were transferred to filter paper and treated with mannitol. Image was taken after 7 days. One representative of three independent experiments (each one including at least thirty plants) is shown.

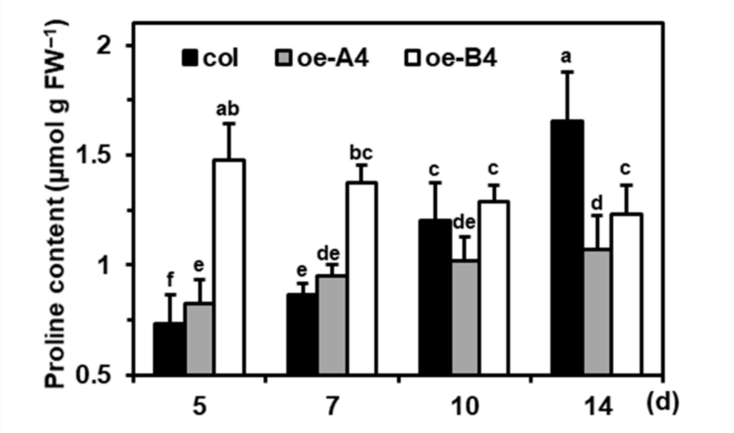

Fig. S9. Determination of proline content of transgenic and wild-type plants at different development stages. The plants at different development stages were collected for determination of proline. Bars are means ± SD of three independent experiments. Different letters indicate significant differences among these lines (Student–Newman–Keuls test, $P < 0.05$).

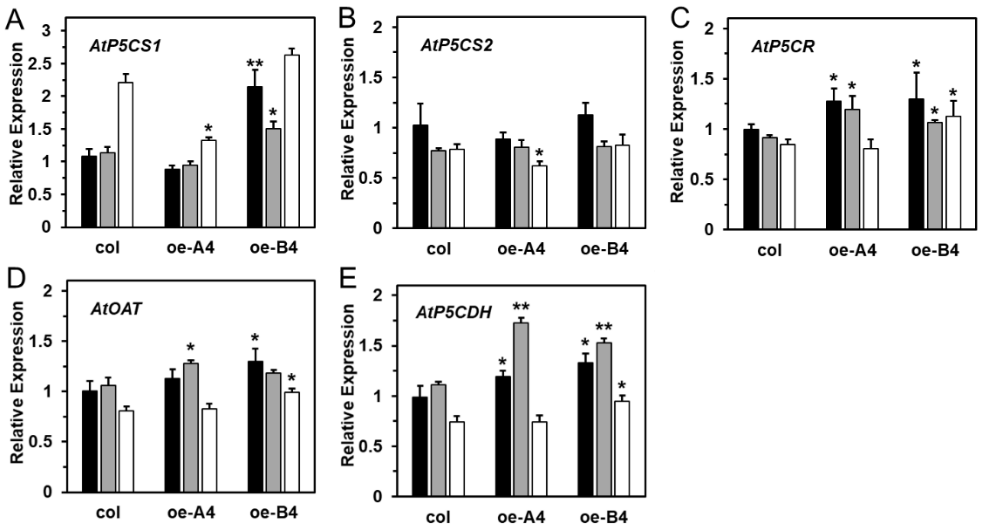

Fig. S10. Analysis of gene expression involved in proline metabolism by qPCR. Fold changes in the expression levels of genes (*AtP5CS1*, *AtP5CS2*, *AtP5CR*, *AtOAT* and *AtP5CDH*) were detected at different stages of the BT treatment by qPCR and with 5-d-old seedlings. H, heat stress. R, recovery. The raw data were normalized by using *AtActin2* as an internal reference. One preventative data set is shown. Bars are means ± SD of three biological replicates. Significant differences between wild-type and transgenic plants are indicated (* $P < 0.05$ and ** $P < 0.01$, Student's *t*-test). *P5CS*, pyrroline-5-carboxylate synthetase; *P5CR*, pyrroline-5-carboxylate reductase; *P5CDH*, P5C dehydrogenase; *OAT*, ornithine-d-aminotransferase.

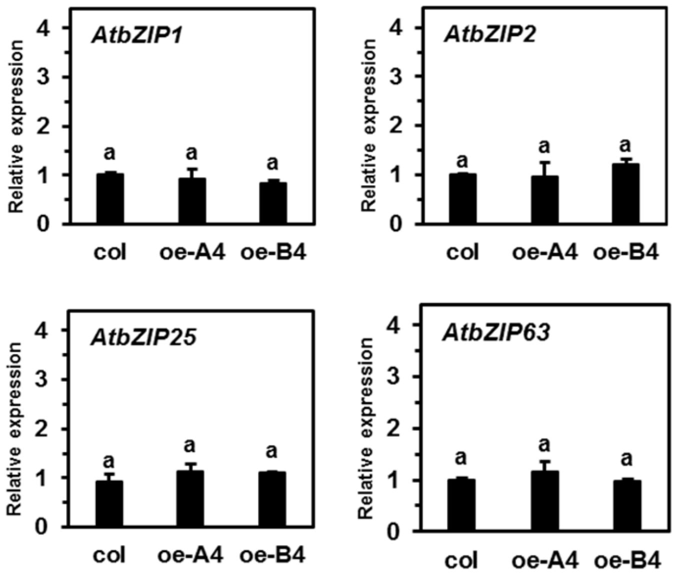

Fig. S11. Analysis of *AtbZIP1*, *AtbZIP2*, *AtbZIP10*, *AtbZIP25* and *AtbZIP63* gene expression by qPCR under normal conditions. 5-day-old seedlings were used for determination. In oe-A4 or oe-B4, some of them was slightly induced, but there were no significant different with wild-type. The raw data were normalized using *AtActin2* as an internal reference. Bars are means ± SD of three biological replicates. The 'a' letter indicate no difference on expression between them (Student–Newman–Keuls test at $P < 0.05$).

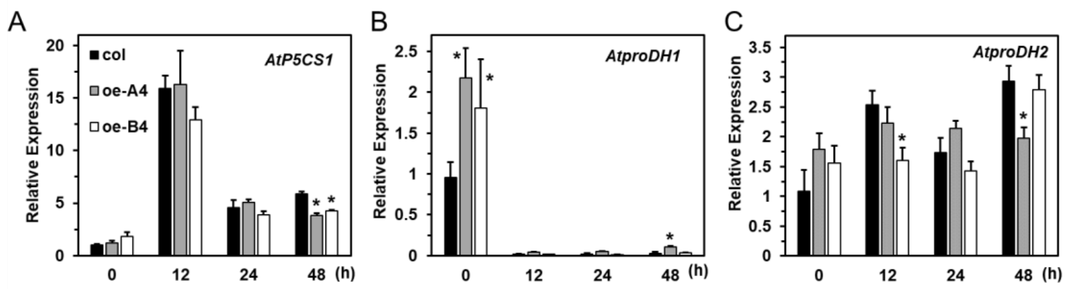

Fig. S12. Analysis of *AtP5CS1*, *AtproDH1* and *AtproDH2* gene expression by qPCR under mannitol stress. Expression of *AtP5CS1, AtproDH1* and *AtproDH2* determined after mannitol treatment by qRT-PCR in wild-type and transgenic lines in absence of sucrose. 6-day-old seedlings transferred to the filter paper, and treated with 300 mM mannitol for 0, 12, 24, and 48 h, then collected for expression analysis. Seedlings were transferred to 1/2-MS liquid medium contained 2% sucrose for 12 h as the 0 h control. The raw data were normalized using *AtActin2* as an internal reference. One preventative data set is shown. Bars are means ± SD of three biological replicates. Significant differences between wild-type and transgenic plants are indicated (* $P < 0.01$, Student's t-test).

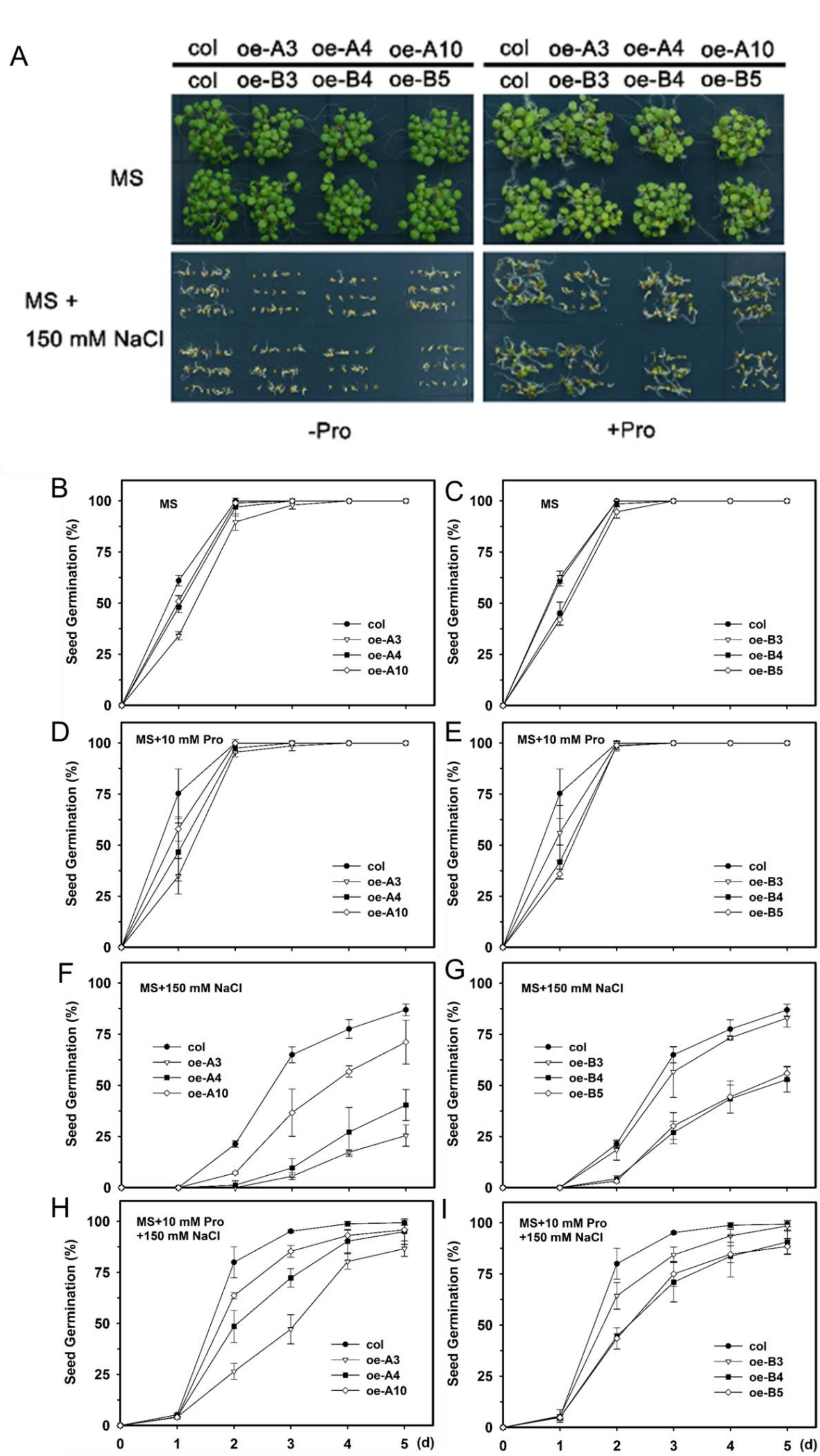
A
col oe-A3 oe-A4 oe-A10
col oe-B3 oe-B4 oe-B5
col oe-A3 oe-A4 oe-A10
col oe-B3 oe-B4 oe-B5
MS
MS +
150 mM NaCl
-Pro
+Pro
B
MS
Seed Germination (%)
col
oe-A3
oe-A4
oe-A10
C
MS
col
oe-B3
oe-B4
oe-B5
D
MS+10 mM Pro
E
MS+10 mM Pro
F
MS+150 mM NaCl
G
MS+150 mM NaCl
H
MS+10 mM Pro
+150 mM NaCl
I
MS+10 mM Pro
+150 mM NaCl
(d)

Fig. S13. Responses of the transgenic plants to salt stress with exogenous proline (Pro). (A) Wild-type and transgenic seeds were sowed in MS medium with 0 or 10 mM proline, and treated with 150 mM NaCl. Image was taken after 5 days. One representative of three independent experiment (each one including over 30 seedlings of each line) is shown. (B-I) Germination potential of these lines under the conditions described in (A). Date represent means ± SD of the germination rate from three independent experiments.
